# Global mRNA decay analysis at single nucleotide resolution reveals segmental and positional degradation patterns in a Gram-positive bacterium

**DOI:** 10.1186/gb-2012-13-4-r30

**Published:** 2012-04-26

**Authors:** Simen M Kristoffersen, Chad Haase, M Ryan Weil, Karla D Passalacqua, Faheem Niazi, Stephen K Hutchison, Brian Desany, Anne-Brit Kolstø, Nicolas J Tourasse, Timothy D Read, Ole Andreas Økstad

**Affiliations:** 1 Laboratory for Microbial Dynamics, Department of Pharmaceutical Biosciences, School of Pharmacy, University of Oslo, PB 1068 Blindern, 0316 Oslo, Norway; 2 Department of Human Genetics, Division of Infectious Diseases, Emory University School of Medicine, 615 Michael Street, Atlanta, GA 30322-1013, USA; 3 Department of Medicine, Division of Infectious Diseases, Emory University School of Medicine, 615 Michael Street, Atlanta, GA 30322-1013, USA; 4 454 Life Sciences, A Roche Company, 1 Commercial Street, Branford, CT 06405, USA; 5 PHARMAQ AS, PO Box 267 Skøyen, N-0213 Oslo, Norway

## Abstract

**Background:**

Recent years have shown a marked increase in the use of next-generation sequencing technologies for quantification of gene expression (RNA sequencing, RNA-Seq). The expression level of a gene is a function of both its rate of transcription and RNA decay, and the influence of mRNA decay rates on gene expression in genome-wide studies of Gram-positive bacteria is under-investigated.

**Results:**

In this work, we employed RNA-Seq in a genome-wide determination of mRNA half-lives in the Gram-positive bacterium *Bacillus cereus*. By utilizing a newly developed normalization protocol, RNA-Seq was used successfully to determine global mRNA decay rates at the single nucleotide level. The analysis revealed positional degradation patterns, with mRNAs being degraded from both ends of the molecule, indicating that both 5' to 3' and 3' to 5' directions of RNA decay are present in *B. cereus*. Other operons showed segmental degradation patterns where specific ORFs within polycistrons were degraded at variable rates, underlining the importance of RNA processing in gene regulation. We determined the half-lives for more than 2,700 ORFs in *B. cereus *ATCC 10987, ranging from less than one minute to more than fifteen minutes, and showed that mRNA decay rate correlates globally with mRNA expression level, GC content, and functional class of the ORF.

**Conclusions:**

To our knowledge, this study presents the first global analysis of mRNA decay in a bacterium at single nucleotide resolution. We provide a proof of principle for using RNA-Seq in bacterial mRNA decay analysis, revealing RNA processing patterns at the single nucleotide level.

## Background

The expression of a gene is subject to numerous levels of regulation; from the initiation of transcription, via RNA processing, translation and degradation of the message, and finally through processing and degradation of the protein product itself. During the past years, it has become apparent that, in addition to transcription initiation, alteration in the rate of messenger decay constitutes an important factor in the regulation of a gene's steady state mRNA expression level [1-4]. In bacteria, several examples are known of altered decay rates for specific mRNAs following changes in cell growth conditions, often mediated by small non-coding RNAs (for examples, see [3-7]). Although the vast majority of *Escherichia coli *mRNAs were reported not to exhibit a highly variable decay rate between different nutrient conditions and growth rates [8], these variables produced altered mRNA half-lives in the Gram-positive bacteria *Lactococcus lactis *and *Streptococcus pyogenes *[5,9]. Several sequence attributes, such as RNase processing sites and the functional classes of the gene products, have been found to influence mRNA decay rate, suggesting that the decay rate is in large part dictated by the mRNA molecule itself [8,10,11].

*E. coli *has long been the preferred model for investigating mRNA decay mechanisms in bacteria. Turnover of mRNA in *E. coli *is performed by a number of enzymes, including some in complexes such as the RNA degradosome, and includes several, and partially redundant, enzymatic activities, including endoribonuclease cleavage, 3'-exoribonuclease, and oligoribonuclease activities, to achieve breakdown to single mononucleotide entities [12,13]. mRNA decay in the Gram-positive model organism *Bacillus subtilis *differs from that of *E. coli *in that several of the key riboendonucleases in these bacteria are different (reviewed by [12]), and only within the last two years has a degradosome-like complex been described in *B. subtilis *[14]. Also, in *B. subtilis *one of the major RNases, RNase J, has 5' to 3' exonuclease activity, an enzymatic activity seemingly absent in *E. coli*. Most RNases, including RNase J and RNase Y, which are thought to be the major RNases in *B. subtilis*, are shared with other Bacilli [15-17]. Indeed, the full complement of key RNases found in *B. subtilis *(RNase Y, RNase J1, RNase J2, RNase III, PNPase, RNase R, RNase PH, RNAse P, RNase Z, RNase HII, MazF/EndoA, YhaM, KapD, RNase HIII, RNase M5, YhcR) [18,19] are present in *Bacillus cereus *(GenBank and UniProt databases). RNase Bsn is also present in a range of *B. cereus *strains, but is, however, absent in the two *B. cereus *strains subject to study here.

*B. cereus *is a Gram-positive spore-forming rod-shaped bacterium that is widely distributed in the environment and may cause food-related disease through two food-poisoning syndromes, emetic or diarrheal. It also constitutes an opportunistic pathogen increasingly being reported as the cause of a range of serious non-gastrointestinal infections, including nosocomially derived bacteremia, wound infections, infections of the central nervous system, and severe endophthalmitis following trauma to the eye (reviewed by [20,21]). *B. cereus *is part of the *B. cereus *group, which embraces six officially recognized species that are closely related [22-24]. More than a hundred *B. cereus *group isolates have been subject to whole genome sequencing (closed or draft), making it one of the groups of bacteria with the highest number of genome sequences available. Despite their genetic similarities, bacteria in this group exhibit different pathogenic specificities toward different hosts, and include *Bacillus anthracis*, the cause of anthrax [25], and entomopathogenic *Bacillus thuringiensis*, the source of the world's most frequently used biological pesticide [26]. Recently, a number of *B. cereus *strains causing anthrax-like disease have been characterized, underlining the close relationship between *B. anthracis *and *B. cereus *and its suitability as a model for certain aspects of *B. anthracis *biology [24,27-29].

During the past decade, microarray technology has been used with great success for global mRNA decay studies in all domains of life [8,10,30-32]. High-density tiling arrays have revealed positional degradation patterns and a 5' to 3' direction of decay for the majority of mRNA transcripts in *E. coli *and cyanobacteria [33,34]. Although high density studies have not yet been conducted in *B. subtilis *(or other Gram-positive bacteria), the major direction of decay is thought to proceed in the 5' to 3' direction [12]. In this paper we present, to our knowledge, the first genome-wide analysis of mRNA decay at single nucleotide resolution using RNA sequencing (RNA-Seq), the emerging state-of-the art technique for global gene expression studies [35,36]. Employing *B. cereus *as a model, we provide genome-wide operon structure predictions for two *B. cereus *strains, mRNA half-lives for more than 2,700 ORFs, and mRNA degradation patterns at single nucleotide resolution for more than 500 operons in *B. cereus *ATCC 10987, a sequenced (closed) model strain that maps to a *B. cereus *group phylogenetic cluster that also encompasses *B. anthracis*.

## Results

### Mapping of sequence reads

Mid-log cultures of *B. cereus *strains ATCC 10987 and ATCC 14579 were subjected to transcriptional arrest by rifampicin (by specific inhibition of bacterial RNA polymerase [12,37]), and RNA was isolated in a time-course series (0, 2.5, 5, and 10 minutes after rifampicin addition). Following rRNA depletion, mRNA levels were quantified by RNA-Seq (Illumina GA-II and Roche 454) as described in the Materials and methods section. Three series of biological replicates, (called 'B', 'C' and 'D') were sequenced for each strain (series B and D using Illumina GA-II, and C and D by 454; Supplementary Table S1 in Additional file 1). Different samples from different time points/series produced a variable number of reads, which was corrected for in the subsequent statistical analyses. Mapping of reads against reference genome sequences was done with Bowtie version 0.11.3 (GA-II data) or Newbler version 2.3 (454 data). Out of a total of 88 and 82 million reads produced by Illumina sequencing from *B. cereus *ATCC 10987 and *B. cereus *ATCC 14579, respectively, 18 million (20.4%) and 12 million (14.6%) reads were unambiguously mapped to each respective genome. For the 454 sequencing data, from a total of 2.1 and 2.5 million sequence reads, 403,000 (19.2%) and 260,000 (10.4%) could be mapped unambiguously in *B. cereus *ATCC 10987 and *B. cereus *ATCC 14579, respectively (Supplementary Table S1 in Additional file 1). The high number of reads that could not be mapped to a single position in a genome largely reflected an incomplete removal of rRNA molecules (Supplementary Table S1 in Additional file 1, columns 10 and 11), the sequences of which can be mapped to several positions in the chromosome due to the presence of 12 and 13 highly similar ribosomal RNA operons, respectively, in the two strains [38,39]. The proportion of unambiguously mapped reads in the C and D series were higher, probably as a result of these series having been subjected to an extra round of rRNA depletion. Interestingly, rRNA constituted most or all of the 454 reads that could not be unambiguously mapped, potentially because the longer 454 reads span other repeated sequences present in these genomes (for example, *bcr1-bcr18*) [40].

### Prediction of transcriptional start sites and operon structures

Operon structures, transcriptional start sites (TSSs) and transcriptional end sites (TESs) for two *B. cereus *strains were resolved by pooling all unambiguously mapped reads from both sequencing technologies (for each strain separately). Although the number of reads from 454 sequencing was lower, the longer sequences (see Materials and methods) helped join operons that terminated in regions for which there was no coverage in the GA-II data. In total, 105 and 228 transcribed units were joined by including the 454 derived sequences, in *B. cereus *ATCC 10987 and *B. cereus *ATCC 14579, respectively. At the point of RNA isolation, the *B. cereus *transcriptome was distributed in 2,271 and 1,696 transcriptional units (TUs) with an average coverage of at least five reads per base pair, in the two strains (Table 1). The operons consisted of 3,685 and 2,061 genes transcribed above the cutoff of 50 reads per kilobase (RPK). The higher numbers in *B. cereus *ATCC 10987 (TUs, expressed genes) probably reflected the higher sequence coverage for this strain. Many operons were monocistronic (1,230 (33.4%) and 1,011 (49.1%), respectively), while 566 (15.4%) and 378 (18.3%) operons contained two ORFs (Supplementary Figure S1 in Additional file 2). Altogether, 27 and 28 operons, respectively, were determined to encompass more than 10 genes. The fractional distribution of gene numbers in operons is similar to what has been presented for *B. subtilis *[41]. The unexpectedly high fraction of monocistronic operons in *B. cereus *ATCC 14579 could be a result of the low number of genes (2,061 relative to 3,685) with sequence coverage above the cutoff (average coverage of at least 5 reads per base pair), relative to *B. cereus *ATCC 10987.

**Table 1:** General features of the *Bacillus cereus *transcriptome, for strains ATCC 10987 and ATCC 14579

	*B. cereus *ATCC 10987	*B. cereus *ATCC 14579
Annotated genes	6,014	5,497
Expressed genes	3,685	2,061
Operons	2,271	1,696
Genes with mRNA half-lives determined	2,745	1,675
TSS determined	1,222	1,002
Median 5' UTR length (nucleotides)	74.5	59
TES determined	918	717
Median 3' UTR length	31	27
Transcribed units with no annotation	20	17
Median gene half-life (minutes)	2.4	2.6
Minimum gene half-life (minutes)	0.6	0.6
Maximum gene half-life (minutes)	15 or above	15 or above
Median gene expression level (RPKM)	44.1	109
Minimum gene expression level (RPKM)	5.2	20.3
Maximum gene expression level (RPKM)	4 × 10^4^	4 × 10^4^

The criterion used for 'expressed genes' was having an expression level higher than 50 RPK in the t0 sample. The criterion used for 'operons' was a continuous stretch of overlapping reads covering one to several genes with the same orientation in the genome and for which the average sequence coverage was higher than five reads per base pair. RPKM, reads per kilobase per megabase.

We have mapped the 5' UTR of 1,222 and 1,002 genes, and the 3' UTR of 918 and 717 genes, in *B. cereus *ATCC 10987 and ATCC 14579 (Table 1; Additional file 3). We identified 34 and 65 TUs in the two strains that had no previous annotation in the RefSeq entries. Out of these, 13 and 17 TUs overlapped with the coordinates of previously characterized RNA elements in the Rfam database [42], and 1 and 4 TUs overlapped with previously identified DNA repeat sequences [40,43]. The removal of TUs with an expression level below 50 RPK left us with 20 and 17 TUs in *B. cereus *ATCC 10987 and ATCC 14579, respectively, for which no previous annotation was available (Additional file 3). By BlastX comparison to the NCBI 'nr' database, two and nine TUs, respectively, matched known proteins with an id% above 50 and at least 70% sequence overlap, and may thus possibly represent unannotated ORFs or pseudogenes. The remaining TUs (18 in ATCC 10987, 8 in ATCC 14579) may represent novel RNA genes. A search of the Rfam database using the WU-BLAST service [44], however, produced no hits to any known RNA sequence. Coordinates for all transcribed units (coding or non-coding) can be found in Additional file 3. In accordance with what has previously been reported [45], we observed that a proportion of annotated pseudogenes is indeed expressed. In *B. cereus *ATCC 10987, 13 out of 32 pseudogenes were expressed above cutoff levels, while in *B. cereus *ATCC 14579, 32 out of 95 pseudogenes showed expression.

**Figure 1:**
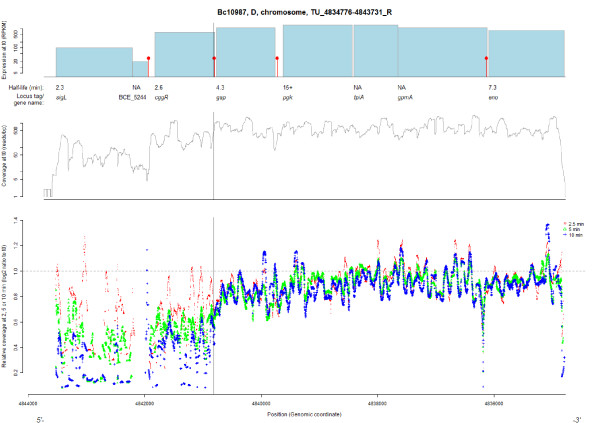
**Degradation pattern for the *gap *operon in *B. cereus *ATCC 10987 at single nucleotide resolution, determined by RNA-Seq**. The upper panel indicates the position of annotated genes in the operon, with the heights of the bars representing the expression values for each gene at t0, and with estimated half-lives and gene names or locus tags given underneath. Transcriptional terminators, as predicted by TransTermHP, are marked by red vertical lines with filled circles. The middle panel shows the coverage per base pair for each nucleotide in the region at t0, while the lower panel shows the log2 ratio of the coverage per base pair for each of the time points following transcriptional arrest, relative to t0 (red, t = 2.5 minutes; green, t = 5 minutes; blue, t = 10 minutes). The *B. cereus gap *operon showed a similar degradation pattern to what has been observed in *B. subtilis *[53]. The solid black vertical line represents the site corresponding to the characterized RNase processing site in *B. subtilis*, where the part of the mRNA encoding the operon regulator CggR is cleaved off and degraded faster than the rest of the operon. Both the position of the RNase cleavage site and the pattern of faster decay of the *cggR *mRNA relative to the rest of the operon seem to be conserved in *B. cereus*. NA, not available.

Rho-independent transcriptional terminators were predicted according to the 'best after gene' algorithm [46] and are marked in Figure 1, and in Additional files 4 and 5. The predicted terminators often coincided with substantial changes in sequence coverage to zero or near zero. Several predicted terminators were, however, located inside operons, and were not associated with large changes in sequence coverage (Figure 1; Additional files 4 and 5), indicating that while the 'best after gene' algorithm is overall quite successful in predicting TESs positioned after the last gene of an operon, it can produce false positive results in *Bacillus *genomes.

In this study, TSSs and TESs were set to coordinates where a shift in coverage from zero to higher than zero was observed (excluding TUs with average sequence coverage lower than 5 reads/bp; see Materials and methods for details). For most of the operons, the determined TSSs and TESs were located at coordinates matching dramatic changes in sequence coverage. The analysis revealed the median 5' UTR length (75 and 59 nucleotides in the *B. cereus *ATCC 10987 and ATCC 14579 strains, respectively) to be in the same range or slightly longer than what was previously reported for the close relative *B. anthracis *[45,47] (median 5' UTR reported to be lower than 40 and 60 nucleotides, respectively) and the less closely related *B. subtilis *[48] (median 5' UTR = 47 nucleotides). We can not exclude that the difference in 5' UTR length between the two *B. cereus *strains is affected by the difference in sequence coverage. The median 3' UTR lengths (31 and 27 nucleotides, respectively) were shorter than the 5' UTRs, and similar to what has been reported both for *B. subtilis *and *B. anthracis *[45,48] (3' UTR = 36 nucleotides or less than 25 nucleotides, respectively). For some operons we observed a coverage of only one or a few reads per base pair directly trailing the predicted TSS, followed by a dramatic rise in coverage some nucleotides downstream, which in these cases most likely represents the correct TSS (Additional file 5). These regions of very low sequence coverage may originate from unspecific polymerization by the RNA polymerase, wrongly mapped sequences, antisense transcription, alternative TSSs, trace amounts of genomic DNA or other biases, and could potentially explain the slight discrepancy between previous studies. To evaluate the TSS predictions, we compared the length of each 5' UTR in the two *B. cereus *strains (this study, Additional file 3) with the length of the 5' UTR for orthologous genes in *B. anthracis*, as determined by Passalacqua *et al*. [47]. Out of 651 orthologous genes with TSS sites determined in both *B. cereus *ATCC 10987 and *B. anthracis *strains, 54% had a 10 bp or less difference in 5' UTR lengths, and 79% differed by 50 bp or less. *B. cereus *ATCC 14579 showed comparable differences in TSS lengths to *B. anthracis *as those described for ATCC 10987 above.

We have previously identified 18 families of intergenic repeated DNA elements in *B. cereus *(*bcr1-bcr18*), of which one was previously suggested to be transcribed (*bcr1*) [40,43,49-51]. In the present study we observed that approximately half of the repeat copies (that is, all elements belonging to the 18 families combined) were expressed, either as part of a 5' or 3' UTR, as a part of an intergenic region within a polycistronic operon, or as part of a transcribed unit with no annotated ORFs; in the two strains, 84 out of 165 (50.9%; *B. cereus *ATCC 10987), and 62 out of 155 (40.0%; *B. cereus *ATCC 14579) intergenic repeats were found to be part of an mRNA molecule. As previously suggested, the high copy number repeat elements *bcr1, bcr2*, and *bcr3 *(structurally and functionally similar to miniature inverted repeat transposable elements (MITEs) [40,50]) were often found to make up parts of UTRs or intergenic regions of operons. However, only the presence of *bcr3 *was shown to correlate with the decay rate of the molecule (see below). Furthermore, *bcr10 *and *bcr18 *elements, which have conserved secondary structures and have been computationally predicted to constitute functional RNA elements, were also transcribed as parts of 5' UTRs, providing further indication that these elements indeed may be regulatory elements working at the RNA level and constitute novel RNAs in the *B. cereus *group [40]. In particular, *bcr18 *is interesting, being found exclusively on plasmids related to *B. anthracis *pXO1 [52], and which thus may constitute a novel regulatory RNA specific to these plasmids.

### Normalization and determination of mRNA decay rates

**Figure 2:**
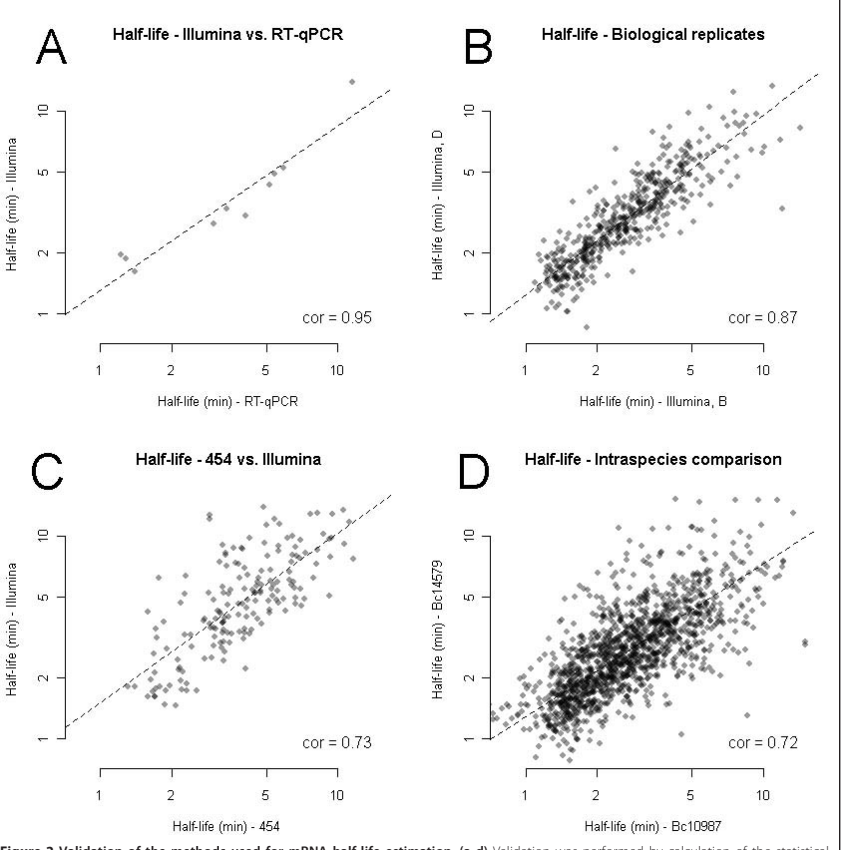
**Validation of the methods used for mRNA half-life estimation**. **(a-d) **Validation was performed by calculation of the statistical correlation between half-lives for corresponding genes determined using different methods (a-c; *B. cereus *ATCC 10987), and statistical correlation between half-lives for orthologous genes from *B. cereus *strains ATCC 10987 and ATCC 14579, respectively (d), determined by RNA-Seq. Half-life values are plotted on log-scale. In each panel, the dashed line gives the regression line between two samples/tests/strains, and the number shown for each plot denotes the Pearson correlation for the gene orthologs. (a) Validation by RT-qPCR of half-lives determined by RNA-Seq (GA-II), demonstrating a good correlation of half-lives determined by the two methods. (b) Correlation between half-lives for the two biological replicate series B and D as determined by RNA-Seq (GA-II). (c) Correlation of mRNA half-lives determined by RNA-Seq, employing GA-II and 454 technology, respectively. (d) Correlation between half-lives of orthologous mRNAs in *B. cereus *strains ATCC 10987 and ATCC 14579, respectively.

To investigate global regulation of mRNA decay in *B. cereus*, we performed a genome-wide determination of mRNA half lives in *B. cereus *ATCC 10987 by RNA-Seq, which measures the half-life of disappearance of sequence (mass) rather than functional half-life. The number of sequence reads produced showed sample to sample variation, possibly caused by variation in sequencing effectiveness, multiple sequencing of some samples, and variable effectiveness of the rRNA depletion procedure. Normalization of the data sets was thus required. As samples were depleted for ribosomal RNA prior to cDNA synthesis (see Materials and methods), possibly with variable efficiency across samples, rRNA could not be used for normalization purposes. We therefore developed a novel normalization protocol, where decay rates for mRNAs from five randomly chosen genes in each strain were determined by reverse transcriptase quantitative PCR (RT-qPCR) and used for normalization (for details, see Materials and methods). To test the validity of the normalization procedure, RT-qPCR was used to determine half-lives for an independent set of mRNAs from another ten randomly selected genes (Figure 2a; list of genes in Supplementary Table S3 in Additional file 1). The analysis produced a Pearson product moment correlation of 0.96 (*n *= 7) and 0.94 (*n *= 9) for the B and D series, respectively, when comparing half-lives estimated by RT-qPCR and RNA-Seq. Furthermore, the half-lives determined using RNA-Seq, for the B- and D-decay series, respectively, showed a correlation of 0.87 (*n *= 493; Figure 2b). The reproducibility of the decay rates, both with different methods and biological replicates, confirmed that RNA-Seq using the presented normalization protocol is a suitable method for mRNA half-life calculations.

The C and D decay series sequenced by 454 technology had less coverage (Supplemental Table S1 in Additional file 1); therefore, it was possible to estimate half-life values only for the most highly expressed genes (46 and 192 genes in the two biological replicates, respectively). There was a reasonable correlation between half-lives determined by RNA-Seq using Illumina and 454 technologies, respectively (Pearson correlation 0.73, *n *= 191; Figure 2c), as well as between RT-qPCR and 454 RNA-Seq (0.76, *n *= 6), although the correlation values were lower than between Illumina data and RT-qPCR. Supplementary Table S3 in Additional file 1 shows the correlation between the genes whose half-lives could be estimated by RT-qPCR and any of the two RNA-Seq methods.

### Genome-wide determination of mRNA decay rates

**Figure 3:**
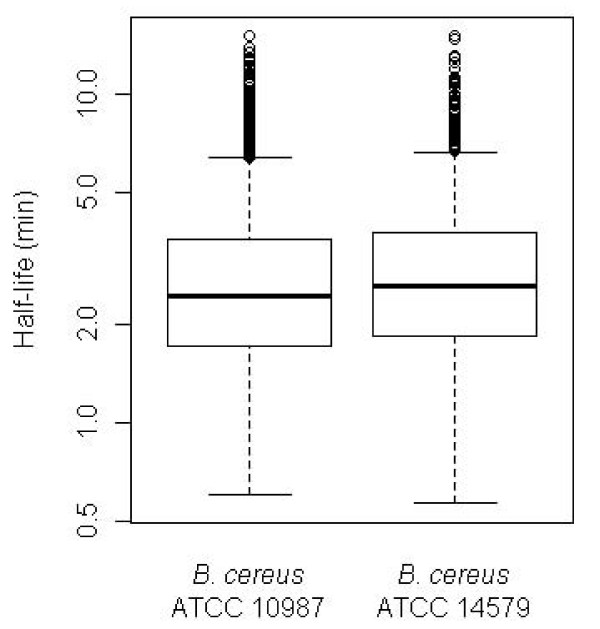
**Distribution of global mRNA half-lives for *B. cereus *ATCC 10987 and *B. cereus *ATCC 14579, respectively, as determined by RNA-Seq using Illumina GA-II technology, and depicted by a boxplot at log scale**. Each box comprises the 25 to 75 percentile of measured mRNA half-lives for the indicated strain (that is, the mid 50% of calculated half-life values). Remaining calculated half-lives (upper and lower 25 percentiles, respectively) are enclosed by whiskers, except for extreme outliers, which are denoted by open circles. The horizontal bar inside each box marks the median mRNA half-life in each of the two bacterial strains (2.4 and 2.6 minutes for *B. cereus *ATCC 10987 and *B. cereus *ATCC 14579, respectively).

Using normalized data from *B. cereus *ATCC 10987 from the Illumina B and D series, mRNA half-lives were determined for 2,745 genes (as described in the previous sections and in Materials and methods), and were found to have median and mean values of 2.4 and 2.9 minutes, respectively (Illumina data only were used to calculate reported half-lives; Figure 3). Half of the estimated half-lives ranged between 1.7 and 3.6 minutes. The shortest half-life observed was 0.6 minutes (BCE_5560, encoding a putative polysaccharide biosynthesis protein), while seven mRNAs showed half-lives longer than 15 minutes (BCE_0700, BCE_3928, and BCE_A0086, encoding orotidine 5'-phosphate decarboxylase, a putative polysaccharide biosynthesis protein, and a prophage protein, respectively, and BCE_4648, BCE_5048, BCE_5241, BCE_A0091, encoding thioredoxin, kinase-associated protein B, phosphoglycerate kinase, and DNA polymerase III beta subunit (beta clamp), respectively). For these genes, half-lives were set to '15 or more', as observations above this value were considered beyond the resolution of the method. Although for the majority of transcripts similar decay rates were observed for each gene within an operon, a substantial number of polycistronic operons exhibited considerable variability in half-lives for component genes. We therefore chose to calculate the half-lives for each ORF within an operon rather than calculating one half-life for each operon as a whole, as the latter could mask biologically relevant differences. For 196 out of the 525 polycistronic transcriptional units mapped in *B. cereus *ATCC 10987, and for which we could estimate half-lives for at least two ORFs, the difference between the minimum and the maximum half-life value for genes within the operon was observed to constitute more than 50% of the average half-life value for the same genes (59 operons with a difference of more than 100%). Comparable numbers were achieved for 290 operons in *B. cereus *ATCC 14579.

To investigate inter-strain variation, we performed a similar mRNA decay analysis for *B. cereus *ATCC 14579 (type strain). Among the two biological replicates analyzed, the D series showed a Pearson correlation of 0.94 with half-lives estimated by RT-qPCR (*n *= 6), which was comparable to what was observed for both biological replicates for *B. cereus *ATCC 10987. The other ATCC 14579 replicate (B series), however, showed a low correlation with RT-qPCR data (Pearson correlation 0.67, *n *= 6), as well as with the ATCC 14579 D series (correlation 0.60; Supplementary Figure S4 in Additional file 2). Based on the analysis of the B and D series, mRNA half-lives could be estimated for 1,675 genes from *B. cereus *ATCC 14579, which was lower than for ATCC 10987, potentially due to a lower sequence coverage. The half-lives showed a distribution similar to that found for *B. cereus *ATCC 10987 (Figure 3). A comparison of mRNA half-lives in the two *B. cereus *strains showed a correlation of 0.72 (Figure 2d) for orthologous genes, probably affected by the poor correlation in the ATCC 14579 B-replicate, and thus in sum a higher uncertainty in half-life values for the ATCC 14579 data.

### High-resolution decay patterns demonstrate specific patterns of transcript degradation

**Figure 4:**
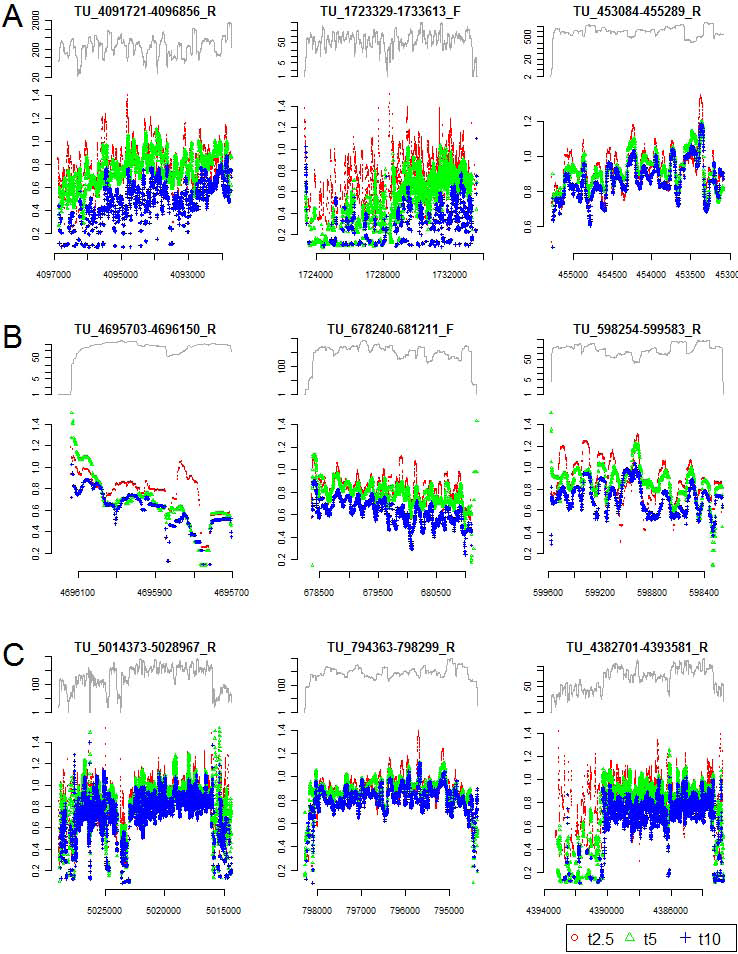
**Representative examples of mRNA decay patterns for selected operons from *B. cereus *ATCC 10897**. For each operon, the upper graph displays the coverage at t0 plotted from the start to the end of the operon, while the lower graph shows, for each single nucleotide position, the log2 ratio of the sequence coverage at different time-points following rifampicin addition (red, t2.5, 2.5 minutes; green, t5, 5 minutes; blue, t10, 10 minutes), relative to the sequence coverage at t0. **(a) **Operons demonstrating examples of 5' to 3' direction of mRNA decay, that is, the sequence coverage for bases near the 5' end of each ORF in the mRNA drops faster than for bases at the 3' end of ORFs. **(b) **Operons demonstrating examples of 3' to 5' direction of mRNA decay. The seemingly anomalous pattern for the 2.5 minute or 5 minute time-points in the 5' end of the TU_4695703-4696150_R transcript (green graph, leftmost panel) may be due to technical noise. **(c) **Operons showing various complex types of decay patterns, where specific blocks of the polycistronic mRNA are degraded faster, or show a pattern of faster decay at both 5' and 3' ends relative to other parts of the mRNA.

In order to obtain a detailed view of messenger stability for operons across the genome, high-resolution degradation patterns were generated for all operons having an average coverage of 50 reads or more per base pair in the t0 sample. Sequence coverage for time-points after t0 were normalized using the same factor that was employed earlier for the estimation of decay rates of individual genes. By plotting the log2 ratio between the coverage of later time-points (t2.5, t5, or t10) and the coverage at the point of transcriptional arrest (t0), a multitude of patterns of transcript degradation were revealed (Figure 4). In total, we investigated degradation patterns at single nucleotide resolution for 571 operons in *B. cereus *ATCC 10987 (of which 347 are polycistronic), and 120 operons in *B. cereus *ATCC 14579 (of which 74 are polycistronic) (Additional file 5). Statistical rank analysis (Spearman *rho*) was employed to investigate the direction of mRNA decay for the various operons analyzed, by calculating the correlation between the log2 ratios for transcript degradation for each ORF, and the distance (in base pairs) from the ORF start. As a range of operons were found to exhibit segmental decay patterns, the analysis was performed on single ORFs rather than on each TU as a whole. In *B. cereus *ATCC 10987, a positive correlation of *rho *> 0.3 (*P*-values < 0.0005) was identified for 324 out of 1,484 ORFs (21.8%; Table 2; some examples shown in Figure 4a), reflecting a 5' to 3' direction of degradation, in accordance with previous observations in *E. coli *and cyanobacteria [33,34]. At 10 minutes following transcriptional arrest, these operons showed an average rank correlation (*rho*) of 0.52 (that is, the log2 ratio to the coverage at t0 being lower near the 5' end), suggesting that, for these molecules, degradation from the 5' end is dominating. This result is further substantiated by the fact that rank correlation increased over time for these ORFs, having an average *rho *of 0.20 and 0.23 at the time-points t2.5 and t5, respectively (Table 2). Also, a number of ORFs seemed to be degraded mainly from the 3' end; for 327 ORFs (22.0%) a rank correlation of less than -0.3 was observed (average -0.54, *P*-values < 0.0005), indicating that the log2-ratio was decreasing with increasing distance from the ORF start (Table 2; Figure 4b). For these operons the average *rho *at t2.5 and t5 was -0.19 and -0.20, respectively. The remaining 833 ORFs (56.1%) had rank correlations between 0.3 and -0.3 at t10, showing no strong tendency towards degradation from any specific end. Similar patterns were obtained in the *B. cereus *ATCC 14579 strain, where 145 (29.9%) and 86 (17.8%) out of 484 ORFs showed mainly 5' to 3' and 3' to 5' directions of decay, respectively. Interestingly, more complex degradation patterns were also observed for a number of operons, for example, transcripts with segmental degradation patterns (Figure 4c).

**Table 2:** Determination of directionality of mRNA decay for *B. cereus *strains ATCC 10987 and ATCC 14579, based on rank statistics (Spearman *rho*)

	N (number of ORFs analyzed)	**5' to 3' direction of decay**^**a**^				**3' to 5' direction of decay**^**a**^			
			
Strain		N	***rho***^**b **^**2.5 minutes**	*rho *5 minutes	*rho *10 minutes	N	*rho *2.5 minutes	*rho *5 minutes	*rho *10 minutes
*B. cereus *ATCC 10987	1,484	324 (22%)	0.2	0.23	0.52	327 (22%)	-0.19	-0.2	-0.54
*B. cereus *ATCC 14579	484	145 (30%)	0.23	0.35	0.51	86 (18%)	-0.15	-0.21	-0.57

^a^The log2 ratio of the coverage at each time-point to the coverage at t0 was calculated, and the rank correlation (Spearman *rho*) between this ratio (for each time-point separately) and the nucleotide number throughout each ORF (that is, distance to the 5' end) was determined. A positive rho signifies that coverage is dropping faster near the 5' end of the molecule, that is, the mRNA is predominantly degraded from the 5' end. For each ORF a *rho *higher than 0.3 or lower than -0.3, and a *P*-value < 0.0005 at t10, was interpreted as 5' to 3' direction of decay or 3' to 5' direction of decay, respectively. ^b^The average *rho *of the ORFs for which a direction of decay could be determined is given, showing that the directionality increases over time.

### Examples of complex degradation patterns in selected operons

To portray some of the more complex degradation patterns observed in the current data set, we have chosen some examples for a more detailed description. Glycolysis is an important energy-generating pathway for many species of bacteria, and the *gap *operon encodes several enzymatic activities that are part of the pathway, including glyceraldehyde-3-phosphate dehydrogenase (GAPDH). Analysis of gene expression level and mRNA half-lives for genes in the *gap *operon from *B. cereus *ATCC 10987 (Figure 1) showed that, as observed previously for *B. subtilis *[53], the 5' part of the polycistronic mRNA covering *cggR*, encoding a transcriptional regulator of the *gap *operon itself, was degraded faster and was less abundant than mRNA from the other genes in the operon. Similarly, a site within *cggR *equivalent to the previously characterized RNase Y processing site in *B. subtilis *corresponded to the position where a significant shift in the log2 ratio coverage was observed in *B. cereus *(Figure 1, solid line), suggesting the presence of a similar processing mechanism in the two species. A similar pattern was observed for the *dnaK *operon, which is known to have a similar regulatory mechanism in *B. subtilis*, where a repressor mRNA, *hrcA*, is cleaved off and degraded at a faster rate [54]. We observed a corresponding pattern for *B. cereus *(see TU_4070529-4083498_R within Additional file 5). Another example of segmental decay is shown in Additional file 4: a locus encoding a ribose operon, where the first four genes, encoding a repressor, a ribokinase, and two subunits of an ATP binding cassette (ABC) transporter, all had lower expression and a shorter half-life than the last three genes, encoding the permease and ribose-binding subunit of the transporter, and a putative transaldolase. It is thus likely that this operon is subject to regulation at the post-transcriptional level. Another example of an ABC transporter with segmental decay pattern is BCE_5508-BCE_5512 (TU_5094641-5100239_R; Additional file 5), where the transport compound (iron) subunit (BCE_5509) of the transporter is degraded at a slower rate than the rest of the genes within the operon. For all the above-mentioned operons, a corresponding decay pattern was observed in both *B. cereus *strains. Several other operons displaying patterns of segmental decay can be found in Additional file 5 (for example, TU_1628067-1635117_F, TU_2126428-2129792_F, TU_4215380-4219707_R, TU_4229502-4238522_R, and TU_4382701-4393581_R).

### Impact of gene function and specific gene sequence attributes on mRNA half-life

**Figure 5:**
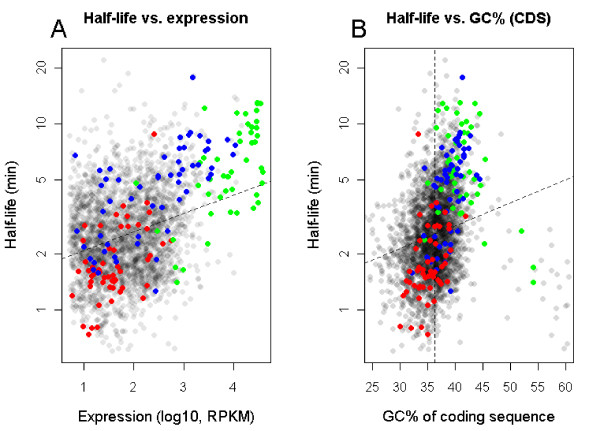
**Statistical correlation between mRNA half-life and gene expression and G+C percentage of coding sequences**. **(a) **Correlation between mRNA half-life and gene expression gene expression. **(b) **Correlation between mRNA half-life and G+C percentage of CDSs (the vertical dashed line marks the mean G+C%). In each panel, the dashed line represents the regression line. mRNA half-lives are plotted at log scale. Genes encoding ribosomal proteins (KEGG 03010) are represented by green dots, genes encoding proteins involved in glycolysis, gluconeogenesis and oxidative phosphorylation (KEGG 00010 and 00190) by blue dots, and genes encoding two component systems by red dots. The data shown are for *B. cereus *ATCC 10987.

To investigate how different parameters of the genes and genome may influence mRNA decay rates and gene expression level, we performed a principal component analysis (PCA) on a large set of variables (Supplementary Table S4 in Additional file 1). Both functional data, such as clusters of orthologous groups (COG) and Kyoto Encyclopedia of Genes and Genomes (KEGG) pathway classification, and specific sequence attributes, such as predicted RNase binding sites, folding energies of secondary structures, and ORF lengths, were tested. ANOVA analysis was carried out in a cyclic manner, so that variance caused by variables other than the one in any particular case under examination was accounted for ahead of *P*-value calculation. Parameters showing a correlation of more than 0.1 with *P*-values below 0.05 were considered significant. A complete list and explanation of the variables tested is presented in Supplementary Table S4 in Additional file 1. The numerical factors most highly correlated with mRNA half-life were expression level (positively) and G+C percentage (positively) of the coding sequence (CDS) (Table 3 and Figure 5). Several non-numerical factors, such as functional class of the gene (COG class), biochemical pathway classification (KEGG), and ribosome binding site (RBS) composition, were also correlated with mRNA half-life (Table 3), as determined by a *post hoc *analysis where half-lives from each category were tested against all other genes with half-lives determined. To account for multiple testing, only *P*-values below 0.005 were accepted as significant, and only classes/factors with more than 25 measurements were accepted (Table 4). By employing either COG or KEGG classification, groups of genes whose products encode proteins involved in metabolism and translation were significantly associated with longer half-lives. Classes containing gene products involved in signal transduction, cell wall biogenesis, genome replication, and ABC transporters were among those with the shortest half-lives (Table 4). In addition, the nucleotide sequence of the ribosomal binding site was correlated with mRNA half-life; using the first five positions of the last six bases in mRNA that are used to base pair with 16S rRNA (as determined by RBSfinder [55]), we found that RBS sequence changes leading to weaker base pairing with rRNA were associated with shorter half-life (Supplementary Figure S5 in Additional file 2). It should be noted, however, that although these differences were statistically significant, the observed changes in half-lives were small (Table 4). Further tests of other groups of genes not described in the COG or KEGG databases revealed that previously identified two-component systems [56] generally had short half-lives (mean half-life 1.8 minutes, *P*-value 10^-7^; Table 4). Other genomic features, such as DNA strandedness, or being plasmid or phage borne, did not show a correlation with mRNA half-life.

**Table 3:** Factors found to be correlated with mRNA half-lives in *B. cereus *ATCC 10987

**Variable**^**a**^	*P*-value	N	Pearson correlation
Expression (RPKM)	10^-15^	2,586	0.33
COG class	10^-08^	2,586	NA
KEGG pathway	0.001	2,586	NA
G+C% coding region	10^-12^	2,586	0.27
Number of genes in operon^b^	10^-5^/0.04/10^-3^	2,586/2421/1847	0.25/0.12/0.10
Second base of RBS	0.02	2,586	NA
Last base of RBS	0.008	2,586	NA
Repeat element part of transcript	10^-18^	2,586	NA

^a^A description of all the different variables is given in Supplementary Table S4 in Additional file 1. ^b^The three numbers represent the data for cases when i) all operons are used, ii) operons composed of more than 20 ORFs are removed, and iii) operons containing more than 5 ORFs are removed. NA, not available.

**Table 4:** Functional gene groups divided into COG classes and their associated mRNA half-lives in *B. cereus *ATCC 10987 (mean within each given class)

**Group/class/factor**^**a**^	*P*-value	Mean half-life (minutes, tested factor)	Mean half-life (minutes, all others)	N (tested factor)
**COG**				
T: signal transduction mechanisms	3.2E-06	2.1	2.6	126
M: cell wall/membrane/envelope biogenesis	6.2E-05	2.1	2.6	141
L: replication, recombination and repair	5.8E-04	2.2	2.6	141
K: transcription	4.0E-04	2.3	2.6	240
Not in COG/no group assigned	4.3E-06	2.3	2.6	586
E: amino acid transport and metabolism	1.2E-03	2.9	2.5	197
G: carbohydrate transport and metabolism	3.7E-03	2.9	2.5	122
H: coenzyme transport and metabolism	2.2E-03	3.0	2.5	91
C: energy production and conversion	1.2E-11	3.7	2.5	129
J: translation, ribosomal structure and biogenesis	< 1.0E-16	3.8	2.5	166
				
**KEGG**				
02010: ABC transporters	5.7E-03	2.3	2.6	81
Not in any pathway	< 1.0E-16	2.4	3.2	2005
00970: aminoacyl-tRNA biosynthesis	2.2E-06	4.0	2.6	30
00010: glycolysis/gluconeogenesis	6.0E-04	4.2	2.6	26
00190: oxidative phosphorylation	1.1E-05	4.4	2.6	30
03010: ribosome	9.5E-12	5.6	2.6	47
				
**RBS, 2nd base**				
Not G: no base pairing with 16S rRNA	5.4E-04	2.2	2.6	150
T: no base pairing with 16S rRNA	1.0E-04	1.9	2.6	30
				
**RBS, last base**				
Not G: no base pairing with 16S rRNA	3.3E-04	2.3	2.6	225
T: no base pairing with 16S rRNA	1.4E-03	2.1	2.6	59
				
**Two-component systems **[56]	1.5E-07	1.8	2.6	53
				
***bcr3*: repeat element part of transcript**	5.6E-04	3.6	2.5	39

^a^A description of all the different variables is given in Supplementary Table S4 in Additional file 1.

Several of the factors that were found to correlate with decay rate, including the G+C content, also correlated with the gene expression level (Table 4). Bias has been reported in Illumina sequencing data for genome regions of variable G+C content; Dohm *et al*. [57] reported an approximate two-fold increase of sequence coverage in areas going from 30% G+C to 40% G+C. To investigate whether a similar sequencing bias could be the reason for the correlation of G+C percentage with gene expression level observed in the present study, we normalized the expression level for each gene with a G+C percentage between 30 and 40 (encompassing 81% of genes in the two genomes; mean G+C% 35.5 and 35.3 for ATCC 10987 and ATCC14579, respectively), by developing an *ad hoc *formula that takes into the account the bias shown in [57]: Exp_adj _= Exp × (G+C% × -0.05 + 2.5). This normalization reduces the expression level of genes with G+C percentage between 30 and 40 at a linear scale, which basically leads to expression levels for genes with a G+C% of 40 being reduced to half of the measured values and the expression of genes with a G+C content of 30% remaining the same. Although we observed a reduction in the *P*-value and in the correlation coefficient, the G+C percentage still retained a significant positive correlation with the gene expression level, suggesting that this effect is biologically relevant. We also tested the 454 dataset for correlation to G+C, and found a non-significant correlation of 0.09. This could, however, be due to the lower sequence coverage in the 454 sample, for which only the 216 highest expressed genes could be tested. Indeed, these genes had a higher G+C% than the average for all genes (39.8% in the 454 data set versus 35.8% for all genes), indicating an over-representation of high G+C percentage within highly expressed genes. To further test this we compared expression values from a microarray experiment performed previously in *B. cereus *ATCC 14579 [58] with the G+C content of the CDS. In accordance with the RNA-Seq analysis, a Pearson correlation of 0.28 was obtained (*P*-value < 10^-16^). Taken together, normalized GA-II expression data, 454 expression data, and microarray expression data all point to the G+C content being a relevant biological factor affecting gene expression. Several other factors correlating with the expression level but not with messenger decay rate were: degree of gene sequence conservation (positively), distance to the chromosomal origin of replication (negatively), belonging to the core genome, being plasmid-borne, and others (Supplementary Figure S6 in Additional file 2 and Supplementary Table S5 in Additional file 1). Interestingly, while genes on pBC10987, a 208 kb circular pXO1-like plasmid *in B. cereus *ATCC 10987 [38,52], are, on average, expressed at a level approximately 10-fold less than genes on the chromosome (Supplementary Table S5 in Additional file 1), the 21 genes on the linear plasmid pBClin15 in *B. cereus *ATCC 14579 [39,59] had an average expression of 1,243 RPKM (reads per kilobase per megabase) during exponential growth, more than 10 times higher than the median whole genome expression (Table 1). These differences can not be explained by a copy-number variance of the respective plasmids, as they have both been estimated to be present in one copy per cell, by coverage depth analysis of DNA sequence data [39,60]. Inclusion of calculated half-lives and expression values from *B. cereus *ATCC 14579 in the PCA generally produced similar results (data not shown).

## Discussion

For the past decade, microarrays have been the tool of choice for genome-wide analyses of mRNA decay. It has been suggested, however, that RNA-Seq will soon replace microarrays as the method of choice for global RNA quantification studies, due to its accuracy, no requirement for prior knowledge of the genome studied, and its unsurpassed resolution [61]. Here we report the genome-wide determination of mRNA half-lives at single nucleotide resolution in the Gram-positive spore former *B. cereus *using RNA-Seq. The analysis additionally revealed operon structures, TSSs and TESs, at a global scale in *B. cereus *bacteria.

### mRNA decay rate estimation using RNA-Seq

By means of a novel normalization method for mRNA decay studies using RNA-Seq data, which should be equally applicable to other bacterial systems, we estimated the half-life for 2,745 genes in *B. cereus *ATCC 10987 and 1,675 genes in *B. cereus *ATCC 14579. The validity of the normalization procedure was confirmed by comparison of half-lives determined by RNA-Seq to half-lives determined by RT-qPCR for an independent set of genes (that is, not used for RNA-seq data normalization; Figure 2a). Analysis of six genes for which half-lives were determined by 454 and Illumina-based sequencing, as well as by RT-qPCR, showed an overall satisfactory agreement by the three methods, although the correlation for the 454 data to RT-qPCR was somewhat lower than for the Illumina data, especially for one gene (BCE4389; Supplementary Table S3 in Additional file 1). In our hands, half-lives determined by RNA-Seq, and in particular those arising from the GA-II data, showed an equal or better correlation with half-lives determined by RT-qPCR, compared to previously observed correlations between results from microarray analysis to RT-qPCR (or to Northern blots, although this is measuring functional half-life) [8,10,32,34,62]. We also experienced good agreement between the two biological replicates from *B. cereus *ATCC 10987 (Figure 2b), in the same range or better than what has been observed for microarrays [8,10]. Further development of RNA-Seq technology for assessment of mRNA half-lives should lead to even greater accuracy for the method in the future. As the cost per base for sequencing is steadily decreasing, one may eventually not need to perform rRNA depletion to economically calculate mRNA transcript abundance, which would allow the use of rRNA for internal normalization instead of quantification by Northern hybridization or RT-qPCR. The use of amplification free RNA-Seq, or improved PCR protocols during Illumina library construction [63], could potentially also increase accuracy and reduce G+C content bias [64,65].

We show here that the half-life of most *B. cereus *mRNA is below 6 minutes, with a median of 2.4 minutes, which is approximately half of what is observed in several other prokaryotes [8,10,32]. A recent study in the cyanobacterium *Prochlorococcus*, however, reports half-lives similar to those reported here [34], and the half-life of bulk mRNA has recently been estimated to be 2.8 minutes in *B. subtilis *[17]. If the difference between *B. subtilis *and *B. cereus *reflects at all a true biological difference, it should be noted that while the present study was performed on cells in the exponential growth phase, the study on *B. subtilis *was done on slower growing cells from stationary phase cultures, which may potentially affect mRNA decay rates. Also, the present study was not performed using strand-specific Illumina sequencing. Thus, antisense RNAs will not be detected, which could affect average global mRNA half-life calculations to some extent. Future studies would greatly benefit from use of strand-specific sequencing, now that methodological biases are well-understood [66].

### Specific patterns of mRNA decay

We obtained single nucleotide resolution degradation patterns for 571 and 120 transcribed units from *B. cereus *strains ATCC 10987 and ATCC 14579, respectively (Figures 1 and 4; Additional files 4 and 5). As previously observed in both *E. coli *and *Prochlorococcus*, most genes seemed to have a slightly faster degradation in the 5' end of the molecule [33,34]. By analysis of the rank correlation for the log2 ratio between the read coverage at the later time-points (t2.5, t5, and t10) and the coverage at t0, we showed that, for a substantial proportion of the mRNAs, the coverage was increasing throughout the molecule, probably reflecting a 5' to 3' direction of decay. Indeed, these findings could support the current hypothesis thought to apply to all domains of life, that mRNA decay is initiated at the 5' end of the RNA, followed by a 5' to 3' direction of decay [12,13,33,34,67,68]. Intriguingly, however, our observations indicate that, in *B. cereus*, almost as many mRNAs exhibit an increasing decay rate from the 3' proximity of the molecule (Table 2; Figure 4b; Additional file 5). This does not necessary rule out the possibility of decay being initiated enzymatically at the 5' end of an endonucleolytically cleaved mRNA, and could potentially be explained by RNase J (or an alternative RNase) binding to sites close to the 3' end of the full-length molecule after an initial conversion of tri- to monophosphate at the 5' end - for instance, due to more proximate site(s) being non-existent, occupied by ribosomes, or otherwise unavailable to RNase binding. It should be noted that although the 5' to 3' direction of decay seems to be predominant in *E. coli*, several other patterns have also been observed [33].

A number of operons with segmental decay patterns were also observed (Figures 1 and 4c; Additional file 4, and several operons within Additional file 5), including the *dnaK *and *gap *operons, which have been thoroughly explored in *B. subtilis *[53,54]. For the *gap *operon, cleaving off of *cggR *is mediated by the recently discovered RNase Y, and a similar pattern was also observed in *B. cereus*, where a significant shift in decay rate was observed at a site corresponding to the RNase Y processing site in *B. subtilis *[14,53,69] (Figure 1; [14,17,53,69]). It is thus tempting to speculate that RNase Y is responsible for the processing of this operon in *B. cereus *as well. These results show that in addition to transcription initiation and termination sites, RNase processing sites in mRNA from Gram-positive bacteria can be conserved through evolution, as has previously been shown for RNase E and its processing sites in tRNA in Gram-negatives [70].

Although several previous global mRNA decay studies have reported half-lives for genes within predicted operons to be generally similar [10,32,62], our observations are in line with other reports of segmental decay patterns within polycistronic operons, originating from global half-life studies utilizing high density microarrays [33,34]. In *B. anthracis*, Martin *et al*. [45] showed that although the majority of gene pairs within an operon had similar expression levels (Pearson correlation 0.85), several pairs of genes demonstrated a marked difference in expression, which could in part be explained by a difference in the decay rates of individual ORFs within the operon. In addition to *gap *and *dnaK*, the previously undescribed ribose operon (Additional file 4) and an ABC transporter (TU_5094641-5100239_R; Additional file 5) showed segmental decay patterns conserved in both *B. cereus *strains analyzed here. These examples, as well as decay patterns observed for a range of operons shown in Additional file 5, all suggest that segmental decay indeed is a widely used mechanism for regulation of transcript levels of individual genes within *B. cereus *polycistrons.

### General trends of mRNA decay

Over the past years, several studies have been published aiming to identify factors determining the rate at which any particular mRNA decays [8,10,32-34,62,71]. As the number of different RNases in a cell is relatively low compared to the number of different mRNAs, it is likely that the decay rates of individual mRNAs are, to some extent, determined by intrinsic factors of the molecule itself [32]. It has recently become evident that the decay of an mRNA molecule is a highly regulated process, and an important factor in regulating the steady state gene expression level [1,2,4]. In the present study, several factors intrinsic to the mRNA molecule itself were investigated for correlation to mRNA decay rate, including G+C content, folding energies in specific parts of the molecule, and composition of and distance to the RBS, several of which were found to be correlated with half-life (Table 3). Bechhofer [12] has suggested that the stability of an mRNA to some degree can be predicted in *B. subtilis*, where, for example, mRNAs with a strong 5' secondary structure close to the RBS, or with an RBS positioned very close to the 5' end of the molecule, generally correspond to transcripts with high stability. This may reflect the protection of the 5' end of the molecule from RNase J attack, either by the secondary structure or by ribosome binding *per se*. Data from *B. cereus *(present study), however, suggest that neither the number of single-stranded nucleotides at the 5' end nor the strength (free folding energy) of secondary structures in the 5' region are globally correlated with mRNA stability (*P*-values > 0.65). Bechhofer's [12] analysis was based partly on mutational studies of single mRNAs, where such differences are more easily detected. However, on a global scale, in *B. cereus*, it seems these are not major general determinants of mRNA stability, in line with what was found globally for *E. coli *[8]. Notably, a correlation has been observed with the strength of the secondary structure in this region and the number of single-stranded nucleotides at the 5' end for single mRNAs in both *E. coli *and *B. subtilis *[72,73] (reviewed in [13]).

In *B. subtilis*, several studies have shown that reduced efficiency of ribosome binding to mRNA leads to an increased mRNA decay rate [73-75]. Accordingly, we observed that genome-wide in *B. cereus*, sequence composition in the RBS-binding site leading to imperfect base pairing with the 3' end of the 16S ribosomal RNA confers a decrease in half-life. Although the correlation was weak, this strengthens the hypothesis that translationally active mRNAs will be protected from the mRNA degradation machinery. We also observed a correlation between the presence of certain co-transcribed intergenic repeat sequences and mRNA half-life, where genes being co-transcribed with *bcr3 *[40,43] had prolonged mRNA half-lives. The relatively small numbers of transcripts carrying these repeats, however, made it difficult to firmly conclude whether it is the presence of the repeat sequences themselves or other factors common to these transcripts that are conferring the observed increase in stability - for example, the fact that genes associated with *bcr3 *are often associated with housekeeping functions [40].

Earlier genome-wide studies have shown that there is a correlation between gene function and mRNA half-life for organisms in all domains of life [8,10,31,76]. In *B. cereus*, we found a significant correlation between COG and KEGG classes of genes and their half-lives, where, for example, genes involved in carbohydrate metabolism and in translation generally had longer half-lives, whereas genes involved in cell wall/membrane/envelope biogenesis, signal transduction, and transport generally had shorter half-lives. In summary, typical housekeeping genes were found to have longer half-lives, which makes sense from a functional and energy-economic perspective. This is, however, in contradiction to what is observed in Archaea, where these classes have an overrepresentation of short-lived mRNAs [10]. Our results are partly in agreement with what is observed in *E. coli*, where gene classes encoding proteins involved in energy metabolism have longer half-lives [8,33], and genes involved in translation are underrepresented among short lived transcripts [33]. It should be noted that several of these studies use different gene function classification systems, with classes spanning a multitude of different subclasses with different functions, and which may be the reason for some of the apparent discrepancy.

In most other global mRNA decay studies, little or a negative correlation between mRNA half-life and the steady state mRNA expression level has been observed [8,10,31]. In the present study, a positive correlation (0.36) between half-life and gene expression was detected. Energy-wise, it would seem to pay off for the cell not to degrade highly expressed mRNAs fast, and a positive correlation between mRNA half-life and the steady-state expression level should intuitively be expected. It should also be noted that although a positive correlation between these factors is observed in *B. cereus*, the variance is high, possibly reflecting a need for the cell to promote fast decay of highly expressed genes that are needed only for short windows in time.

## Conclusions

Use of RNA-Seq revealed a complex mRNA degradation pattern for many operons in *B. cereus*, showing that RNA processing indeed constitutes an important regulatory mechanism governing mRNA expression levels. This study provides a proof-of-principle that RNA-Seq can be used for genome-wide studies of mRNA decay. We expect that this method will be further employed in decay studies in the near future, possibly in combination with 5' and 3' end tagging or chromatin immunoprecipitation sequencing (ChIP-Seq) experiments that would promote discovery of RNase processing sites, or in combination with knock-out studies to explore detailed functional aspects of different RNases in a variety of microorganisms.

## Materials and methods

### Bacterial growth and RNA isolation

*B. cereus *strains ATCC 10987 and ATCC 14579 were grown to mid-exponential phase (optical density = 0.60) in Luria-Bertani (LB) broth (500 ml) at 30°C, 250 rpm. Rifampicin (10 ml) suspended in 5% DMSO/95% LB medium was added to a final concentration of 150 μg/ml for transcriptional arrest. Samples (20 ml) for mRNA isolation were drawn at 0, 2.5, 5, and 10 minutes following rifampicin addition, immediately suspended in an equal volume of methanol/phenol (50:1), and incubated 3 to 5 minutes at room temperature, before harvesting of cells by centrifugation (4,000 × g, 4°C, 5 minutes). Cell pellets were snap-frozen in liquid nitrogen and stored at -80°C. For RNA isolation, cell pellets were suspended in RLT buffer (Qiagen, Hilden, Germany) and lysed using 0.1 μm glass beads in a Precellys 24 instrument at 5,800 rpm for 30 s × 2 (Bertin Technologies, Saint Quentin en Yvelines, France). RNA was isolated using the RNeasy Mini kit (Qiagen) according to the manufacturer's protocol, including the optional on-column DNase treatment. Isolated RNA was treated with Turbo DNase (Ambion, Grand Island, New York, USA) and subjected to clean-up using the RNeasy Mini kit. RNA integrity was investigated by agarose gel electrophoresis, and RNA purity by UV absorbance measurements at 260 and 280 nm.

During the past years a different strategy has been employed for RNA decay experiments, involving the use of 4-thiouracil and the enzyme uracil phosphoribosyltransferase (UPRT) in pulse (-chase) labeling experiments, thereby avoiding the use of antibiotics such as rifampicin to study RNA decay [77-79]. The procedure, originally developed by Cleary and co-workers [80], however requires that the organism under investigation does not encode its own UPRT enzyme or other enzymes that efficiently convert uracil to uridine-monophosphate (UMP) [81]. Many bacteria encode UPRT activities in their genomes, including the bacteria in the *Bacillus cereus *group (*Bacillus cereus *ATCC 10987: Q72XD8, *B. cereus *ATCC 14579: Q814V3). 4-Thiouracil pulse-chase experiments can thus not be expected to work for these organisms, making the rifampicin-based approach the method of choice for genome-wide studies of RNA decay [12].

### RT-qPCR

RT-qPCR was performed by reverse transcription of 1 or 2 μg total RNA (not depleted for rRNA) using random hexamers (Applied Biosystems, Carlsbad, California, USA) and Superscript III (Invitrogen, Grand Island, New York, USA), following the manufacturer's instructions. qPCR was performed on 3 μl 10× diluted cDNA (for gene-specific primers) or 1.5 μl of 10^4^× diluted cDNA (for 16S rRNA primers), employing a final concentration of 2 μM of each primer, and enzyme and reaction buffer supplied by the manufacturer, using either the qPCR-&GO kit (MP Biomedicals, Solon, Ohio, USA) or the LightCycler^® ^480 DNA SYBR Green I Master kit (Roche, Mannheim, Germany). mRNA half-lives were calculated as described below, with expression levels determined using the ΔΔCt-method and normalized using 16S rRNA as reference. The use of 16S rRNA as reference for the RT-qPCR-based mRNA decay experiments was validated by measuring transcript decay (using identical amounts of RNA from the same amount of cells) in the 10 minute window following rifampicin addition, confirming that 16S rRNA is not subject to detectable degradation in this time window (Supplementary Figure S7 in Additional file 2). Primer sequences are available on request.

All samples used in the RNA-Seq experiments were subject to qPCR, with the 'no reverse transcriptase control' producing no products, or very low amounts of product with C_t _values in the range of 35 to 40. Typically, these products had melting temperature (T_m_) values different from the target product, and probably constituted primer dimers, altogether confirming the absence of genomic DNA contamination in the RNA samples.

### RNA-Seq - cDNA library preparation and sequencing

rRNA was depleted using one (B series) or two (C and D series) rounds of treatment with the Microbe Express kit (Ambion) following the manufacturer's instructions. The Microbe Express kit is developed for *E. coli*, and only a portion of the rRNA was removed from the *B. cereus *total RNA. Subjecting the total RNA to an extra round of rRNA depletion, however, significantly improved the depletion efficiency, increasing the proportion of unambiguously mapped reads more than four-fold (Supplementary Table S1 in Additional file 1). Typically, one microgram of RNA was recovered from ten micrograms of input RNA after two rounds of depletion, and used for cDNA synthesis.

For Illumina GA-II sequencing, cDNA was prepared using the SuperScript II Double Stranded cDNA synthesis kit (Invitrogen). Libraries for sequencing on the Illumina GA-II were prepared using 5 μg input cDNA (following Illumina standard protocols), with an insert (fragment) size of approximately 250 nucleotides. Following cluster generation, libraries were sequenced in one direction for 52 cycles or from both ends of the fragment (paired-end sequencing) for 36 cycles each.

For 454 sequencing, 200 ng of input RNA was used to generate cDNA and subsequently prepare 454 GS Junior or GS FLX Titanium sequencing libraries according to the Roche/454 cDNA Rapid Library Preparation protocol. Libraries were sequenced using either the 454 GS Junior or 454 GS FLX Titanium instrument, producing median read lengths of 354 bp for *B. cereus *ATCC 10987 and 438 bp for *B. cereus *ATCC 14579 (box plots of read length distributions are found in Supplementary Figure S8 in Additional file 2).

### Mapping of cDNA sequencing reads

Illumina sequencing reads were mapped to the respective *B. cereus *genomes (RefSeq accession numbers; NC_003909, NC_005707, NC_004721, and NC_004722) with Bowtie 0.11.3 [82] allowing for three mismatches in the two-thirds highest quality part of the sequence ('-v 3 -q --solexa1.3-quals' flags). Paired-end reads with a gap shorter than 18 nucleotides or longer than 300 nucleotides were discarded ('-I 18 -X 300' flags; the average gap size for paired-end reads in the data set was determined to be 174 nucleotides, corresponding to an average fragment length of 246 nucleotides). Reads that could be mapped to several positions in the genome were assigned to the position that was most similar (or identical) (--tryhard --best --strata flags). Reads that, under the specified criteria, could not be assigned an unambiguous position were discarded ('-m 1' flag). Reads mapping to rRNA loci were identified as above, but by using the '-m 20' flag (identifying reads mapping up to 20 times in the genome), and then by comparing mapping coordinates to the coordinates for rRNA loci in the RefSeq entries (accession numbers specified above). An overlap of 50% or more between coordinates was scored as a match. 454 sequencing reads were mapped using the transcriptome mapper tool ('-cDNA' flag) of the Newbler software package (version 2.3) with default settings. Technical replicates (both single and paired) were pooled prior to mapping, and reads that could be mapped to several genome positions were discarded. 454 reads mapping to rRNA loci were identified as above, by mapping to extracted DNA sequences from the complete rRNA loci directly (RefSeq entries as specified above).

### Transcriptional start and end sites, and operon predictions

Under the assumption that the TSSs and TESs should not differ between biological replicates, we pooled all unambiguously mapped reads from all time-points and both sequencing methods, and determined the read coverage of each base in the respective genomes using a Perl script developed by Passalacqua *et al*. [47]. From this data set, operon structures, and TSSs and TESs were determined using an in-house Perl script (Additional file 6) according to the following set of rules: all continuous stretches of sequence coverage with an average coverage above five reads/bp were marked as a TU. TUs that were separated by 5 bp or less were fused. We further mapped, by sequence analysis, all non-unique 52-mers in the *B. cereus *genomes that could not be covered by 52 bp reads (from GA-II single run) due to their ambiguous nature. Adjacent TUs starting and ending within the same non-unique 52-mer (or in several non-unique 52-mers directly following each other) were fused. The TUs were then associated with gene coordinates according to the following rules: i) units that started before and ended after one or more genes in the same direction were defined as a transcribed region; ii) for TUs starting 3' of the start of a coding region or ending 5' of a gene's stop codon, a tag named 'transcription start site (TSS) inside gene', or 'transcription end site (TES) inside gene' was added, respectively; iii) TUs covering two or more genes in opposite orientations were split. For oppositely oriented genes with facing 5' ends the TSS was set to the annotated gene start site, while for oppositely oriented genes with facing 3' ends the TES was set to the annotated gene stop site, and flagged accordingly. Thus, some 5' and 3' ends will be missing in the data set, from transcripts from convergently or divergently transcribed genes that were predicted to be within the same TU.

### Quantification of gene expression

Gene expression levels were determined with the SeqMonk program, version 0.6.1 [83]. The gene expression level was defined as the total number of reads mapped to the CDS, multiplied by 1,000, and divided by the length of the CDS (in base pairs), which gives RPK of CDS. To determine the expression level of each gene at the time of rifampicin addition (t0), the RPK was also adjusted for the total number of unambiguously mapped reads in each sample (RPK-value × 10^6 ^divided by the number of reads), giving the RPK per million base pairs (RPKM) value. Technical replicates (both paired and single) were pooled for each sample.

### mRNA half-life analysis

In order to adjust for unequal amounts of cDNA in each sample and the expected drop in mRNA expression levels during the 10 minute time-course, as well as for potentially variable efficiency of the rRNA depletion procedure performed prior to cDNA synthesis, expression data were normalized for each time point after t0 by employing a novel method that adjusts for varying amounts of RNA in samples ahead of cDNA synthesis, and does not involve simple spiking of samples, which has been used in other studies [77]. mRNA half-life was determined by RT-qPCR for five randomly chosen chromosomal genes (BCE_0110/BC0131, BCE_0518/BC0447, BCE_3851/BC3811, BCE_3877/BC3833, and BCE_5623/BC5474), by using non-rRNA-depleted samples from time points t0 (time of rifampicin addition), t2.5, t5 and t10, and by using 16S rRNA for normalization. The mean of at least three biological replicates was used (see 'RT-qPCR' section above). The selected genes spanned a spectrum from being part of a predicted bicistronic operon, a 13-gene operon, and 3 operons of more than 20 genes. Half-lives for the genes varied between 3.1 minutes and 17.8 minutes, and their abundance at t0 spanned between 100 and 30,095 RPKM. Using the calculated mRNA half-lives for the five genes (from RT-qPCR), and RNA-Seq data for the same five genes from each biological replicate, allowed us to calculate normalization factors for each time point at which RNA was isolated, for each replicate, by fitting to the calculated mRNA decay curve for each gene (Supplementary Figure S9 in Additional file 2). For each biological replicate, the geometric mean of the five normalization factors produced for the set of genes for each of the time-points t2.5, t5 and t10 was used to normalize expression (RPK) values for all remaining genes in the genome at each of the time points. The geometric mean was chosen, as this controls better for abundance differences between different genes and weighing down outlier values [84]. Half-lives were then determined by fitting each of the normalized RPK values for the time-points 0, 2.5, 5, and 10 minutes following rifampicin addition to an exponentially falling curve as previously described [85]. Genes with an expression level < 50 RPK in t0 were excluded, as were genes that were fitted to the curve with a squared correlation coefficient (R^2^) value < 0.8, or which had data missing for more than one time point after t0. The exclusion from the analysis of genes with lower expression levels was done to obtain satisfactory statistical support for estimated half-lives, but will probably introduce some degree of bias to calculations of genome-wide average mRNA half-life and abundance values. Genes with an estimated half-life longer than 15 minutes or having a negative half-life (increase in transcript number during the time-course following rifampicin addition) had their half-life set to 15 minutes or over. The *B. cereus *ATCC 14579 series and all time series determined with 454 sequencing technology were normalized accordingly.

### Statistical and bioinformatics analyses

All statistical analyses were performed in R [86]. PCA was carried out using the 'survival' package [87], version 2.35-8, of R. Several possible variables for each gene were tested for correlation with its half-life and expression (all variables are listed in Supplementary Table S4 in Additional file 1). The PCA was carried out using ANOVA with truncated data, where all half-lives above 15 were set to 15 or over. The ANOVA was carried out in a cyclic manner, so that variance caused by variables other than the one in any particular case under examination was accounted for ahead of *P*-value calculation. All calculations were performed on log-transformed half-life and expression level values, and the distribution of values for each tested factor was scaled by the standard deviation to obtain even variance for all the different factors.

RNA secondary structures in 40 bp windows associated with start of CDSs, RBSs, TSSs and 3' UTRs were predicted by computational folding as linear RNA molecules by means of MFOLD 3.1 [88]. The most stable structure was selected for each sequence.

Potential RNase binding sites were predicted by extracting all possible 40 mers (both strands) from the *B. cereus *ATCC 10987 and ATCC 14579 genomes, and folding each 40 mer in MFOLD 3.1. Stem-loops were then binned according to folding energy (lower than -5 kcal/mol, lower than -7 kcal/mol, lower than -9 kcal/mol and lower than -11 kcal/mol), length of extracted downstream region (7, 9 or 11 nucleotides downstream were extracted), and finally according to number of GC nucleotides in the extracted region (0, 1, or 2 G or C), altogether producing 36 groups of stem-loops. For each group, stem-loops were mapped back onto the genome as predicted RNase binding sites, and the number of potential RNAse binding sites per gene was investigated for correlation with mRNA half-life by means of PCA.

Transcriptional terminators were predicted using the TransTermHP program [46]. Pairwise sequence alignment of repeats and genes was done according to the Needleman-Wunsch algorithm using Needle from the EMBOSS package [89,90]. Orthologs between the two *B. cereus *strains were detected by reciprocal best Blast-hits, essentially as previously described [91,92]; protein coding genes were detected with BlastP and BlastN [92], while pseudogenes and RNA genes were detected using BlastN [92]. No conflict was observed between BlastP and BlastN results. Unannotated TUs were examined using BlastX [92]. Bioinformatics analyses were carried out using computing services available at the Norwegian EMBnet node [93].

### Data availability

Raw data files have been submitted to the EBI MAGE-TAB database [94], and are available at the ArrayExpress website [95] under accession number E-MTAB-450.

## Abbreviations

ABC: ATP binding cassette; ANOVA: analysis of variance; ATCC: American Type Culture Collection; bp: base pair; CDS: coding sequence; COG: clusters of orthologous groups; KEGG: Kyoto Encyclopedia of Genes and Genomes; ORF: open reading frame; PCA: principal components analysis; RBS: ribosome binding site; RNA-Seq: RNA sequencing; RPK: reads per kilobase; RPKM: reads per kilobase per megabase; RT-qPCR: reverse transcriptase quantitative polymerase chain reaction; TES: transcriptional end site; TSS: transcriptional start site; TU: transcriptional unit; UPRT: uracil phosphoribosyltransferase; UTR: untranslated region.

## Competing interests

The authors declare that they have no competing interests.

## Authors' contributions

Conceived and designed the experiments: SMK, ABK, BD, TDR, OAØ. Performed the experiments: SMK, CH, MRW, KDP, FN, SKH. Analyzed the data: SMK, NJT, TDR, OAØ. Contributed reagents/materials: ABK. Wrote the paper: SMK, KDP, BD, NJT, TDR, OAØ. All authors read and approved the final manuscript.

## Supplementary Material

Additional file 1 **Supplementary Tables S1, S3, S4, and S5**. Supplementary Table S1: overview of raw data and mapping of sequence reads to the respective genomes. Supplementary Table S3: comparison of half-life values estimated by RNA-Seq (GA-II, 454) and RT-qPCR. Supplementary Table S4: explanation of each of the different factors tested in the PCA analyses. Supplementary Table S5: PCA analysis of gene expression level. Click here for file

Additional file 2 **Supplementary Figures S1, S4, S5, S6, S7, S8, and S9**. Supplementary Figure S1: distribution of the number of ORFs within operons. Supplementary Figure S4: statistical correlation (Pearson correlation) of calculated mRNA half-lives for the *B. cereus *ATCC 14579 B series and D series, respectively. Supplementary Figure S5: correlation between the RBS sequence and mRNA half-life. Supplementary Figure S6: correlation between mRNA expression level and selected factors. Supplementary Figure S7: expression of 16S rRNA at 2.5, 5, and 10 minutes after transcriptional arrest by rifampicin, relative to 16S rRNA expression at t(0) (time-point of rifampicin addition). Supplementary Figure S8: distribution of mapped read lengths from RNA sequencing using 454 technology. Supplementary Figure S9: explanation of the normalization procedure for RNA-Seq data using RT-qPCR. Click here for file

Additional file 3 **Gene half-lives and expression values**. Listing of half-life and expression values for each gene in the genomes of *B. cereus *strains ATCC 10987 and ATCC 14579, and coordinates for all transcribed units. Click here for file

Additional file 4 **mRNA degradation patterns for the *rbs *(ribose utilization) operon in *B. cereus *ATCC 10987**. Supplementary Figure S3 showing detailed mRNA decay patterns for the *rbs *operon. Click here for file

Additional file 5 **Global mRNA decay patterns at single nucleotide resolution**. Detailed mRNA decay patterns of highly expressed operons. Click here for file

Additional file 6 **Scripts employed in analyses**. Lists the scripts used for TSS, TES, and operon prediction. Click here for file
