# Supplementary material for: Global mRNA decay analysis at single nucleotide resolution reveals segmental and positional degradation patterns in a Gram-positive bacterium
Source: Genome Biol. 2012 Apr 26;13(4):R30. doi: 10.1186/gb-2012-13-4-r30 (PMC3446304; doi:10.1186/gb-2012-13-4-r30)
Supplement: Additional file 1 — Supplementary Tables S1, S3, S4, and S5. Supplementary Table S1: overview of raw data and mapping of sequence reads to the respective genomes. Supplementary Table S3: comparison of half-life values estimated by RNA-Seq (GA-II, 454) and RT-qPCR. Supplementary Table S4: explanation of each of the different factors tested in the PCA analyses. Supplementary Table S5: PCA analysis of gene expression level. [file gb-2012-13-4-r30-S1.PDF]

Suppl. Table S1: Overview of raw data and mapping of reads to the genomes

Strain	Sequencing technology	Biological replicate	Time-point	Run type (Single / Paired	Total number of reads	Number of unambiguously mapped reads [a]	% unambiguously mapped reads [a]	% reads that could not be mapped [b]	% reads that could not be unambiguously mapped [c]	% reads that map to rRNA	Raw data file(s) [d]
B. cereus ATCC 10987	Illumina GS-II	D	t0	Single	1.27E+07	4.58E+06	36%	5%	59%	54%	10D0_s_7_sequence.fastq.gz
				Paired	1.67E+07	5.06E+06	30%	14%	55%	50%	10D0_s_1_1_sequence.fastq.gz;10D0_s_1_2_sequence.fastq.gz
			t2.5		1.08E+07	1.89E+06	18%	4%	78%	70%	10D2.fastq.gz
			t5	Single	1.50E+07	3.48E+06	23%	3%	74%	69%	10D5.fastq.gz
			t10		1.55E+07	2.05E+06	13%	3%	84%	78%	10D10.fastq.gz
		B	t0	Paired	7.92E+05	5.47E+04	7%	4%	89%	77%	10B0_090810_s_6_1_sequence.fastq.gz;10B0_090810_s_6_2_sequence.fastq.gz
				Single	6.33E+06	4.21E+05	7%	9%	84%	73%	10B0_090924_s_1_sequence.fastq.gz
			t2.5	Paired	4.89E+05	2.98E+04	6%	4%	90%	80%	10B2_090810_s_6_1_sequence.fastq.gz;10B2_090810_s_6_2_sequence.fastq.gz
				Single	3.92E+06	2.21E+05	6%	12%	82%	73%	10B2_090924_s_3_sequence.fastq.gz
			t5	Paired	4.89E+05	1.00E+04	2%	4%	94%	83%	10B5_090810_s_6_1_sequence.fastq.gz;10B5_090810_s_6_2_sequence.fastq.gz
				Single	2.41E+06	5.25E+04	2%	16%	82%	72%	10B5_090924_s_6_sequence.fastq.gz
			t10	Paired	1.85E+06	6.86E+04	4%	3%	93%	83%	10B10_090810_s_6_1_sequence.fastq.gz;10B10_090810_s_6_2_sequence.fastq.gz
					7.31E+06	2.40E+05	3%	3%	94%	83%	10B10_091016_s_7_1_sequence.fastq.gz;10B10_091016_s_7_2_sequence.fastq.gz
	Roche 454	C	t0		7.05E+04	7.18E+03	10%	1%	89%	89%	10c00_1_run1.sff
			t2.5		7.42E+04	5.95E+03	8%	1%	91%	91%	10c02_1_run1.sff
			t5	Single	9.80E+04	1.47E+04	15%	1%	84%	84%	10c05_1_run1.sff
			t10		1.04E+05	1.25E+04	12%	1%	87%	87%	10c10_1_run1.sff
			t20		9.63E+04	5.38E+03	6%	0%	94%	94%	10c20_1_run1.sff
		D	t0		3.17E+05	5.52E+04	17%	1%	82%	82%	10d00_1_run1.sff;10d00_1_run2.sff;10d00_1_runJR.sff;10d00_2_run2.sff
			t2.5		4.33E+05	5.49E+04	13%	1%	86%	86%	10d02_1_run1.sff;10d02_1_run2.sff;10d02_1_runJR.sff;10d02_2_run2.sff;10d02_2_runJR.sff
			t5	Single	3.09E+05	3.07E+04	10%	1%	89%	89%	10d05_1_run1.sff;10d05_1_run2.sff;10d05_1_runJR.sff;10d05_2_run2.sff
			t10		3.47E+05	1.98E+05	57%	1%	42%	42%	10d10_1_run1.sff;10d10_1_run2.sff;10d10_2_run2.sff
			t20		3.16E+05	1.78E+04	6%	1%	94%	93%	10d20_1_run1.sff;10d20_1_run2.sff;10d20_1_runJR.sff
	**Both***	**ALL**	ALL	BOTH	9.65E+07	1.86E+07	19%	6%	74%		

Strain	Sequencing technology	Biological replicate	Time-point	Run type (Single / Paired	Total number of reads	Number unambiguously mapped reads [a]	% unambiguously mapped reads [a]	% reads that could not be mapped [b]	% reads that could not be unambiguously mapped [c]	% reads that map to rRNA	Raw data file(s) [d]
B. cereus ATCC 14579	Illumina GS-II	D	t0	Single	1.27E+07	2.47E+06	19%	9%	72%	49%	14D0.fastq.gz
			t2.5		1.42E+07	3.70E+06	26%	3%	71%	54%	14D2.fastq.gz
			t5		1.14E+07	2.88E+06	25%	7%	68%	51%	14D5_s_8_sequence.fastq.gz
			t10		5.42E+06	1.27E+06	23%	9%	68%	50%	14D10_feb_10.fastq.gz
		B	t0	Single	1.05E+07	1.03E+06	10%	13%	78%	62%	14B0_090924_s_2_sequence.fastq.gz
			t2.5	Paired	1.26E+06	2.78E+04	2%	5%	93%	81%	14B2_090810_s_6_1_sequence.fastq.gz;14B2_090810_s_6_2_sequence.fastq.gz
				Single	9.50E+06	3.04E+05	3%	13%	84%	71%	14B2_090924_s_4_sequence.fastq.gz
			t5		9.14E+06	5.33E+05	6%	11%	83%	71%	14B5_090924_s_7_sequence.fastq.gz
			t10	Paired	1.55E+07	1.53E+05	1%	5%	94%	82%	14B10_091016_s_8_1_sequence.fastq.gz;14B10_091016_s_8_2_sequence.fastq.gz
	Roche 454	C	t0	Single	9.85E+04	2.67E+04	27%	1%	72%	73%	14c00_1_run1.sff
			t2.5		8.91E+04	7.35E+03	8%	1%	91%	92%	14c02_1_run1.sff
			t5		6.53E+04	4.62E+03	7%	0%	92%	94%	14c05_1_run1.sff
			t10		6.47E+04	1.39E+03	2%	0%	97%	98%	14c10_1_run1.sff
			t20		4.40E+05	7.68E+03	2%	0%	98%	99%	14c20_1_run1.sff;14c20_2_run1.sff;14c20_3_run1.sff;14c20_4_run1.sff
		D	t0	Single	3.18E+05	6.97E+04	22%	2%	76%	78%	14d00_1_run1.sff;14d00_1_run2.sff;14d00_1_runJR.sff
			t2.5		2.80E+05	5.58E+04	20%	1%	79%	80%	14d02_1_run1.sff;14d02_1_run2.sff;14d02_1_runJR.sff
			t5		5.07E+05	4.29E+04	8%	1%	91%	92%	14d05_1_run1.sff;14d05_1_run2.sff;14d05_1_runJR.sff;14d05_2_run2.sff
			t10		2.91E+05	3.02E+04	10%	1%	89%	90%	14d10_1_run1.sff;14d10_1_run2.sff;14d10_1_runJR.sff
			t20		4.03E+05	1.42E+04	4%	2%	95%	97%	14d20_1_run1.sff;14d20_1_run2.sff;14d20_1_runJR.sff;14d20_2_run2.sff
	**Both***	**ALL**	ALL	BOTH	9.21E+07	1.26E+07	14%	8%	79%		

[a] Reads that could be mapped to a single position in the genome

[b] Percentage of reads that could not be mapped to the genome

[c] Percentage of reads that could be mapped to several positions in the genome, and were therefore removed from the analysis

[d] Name of the raw data files, available in the ArrayExpress Archive (www.ebi.ac.uk/arrayexpress) under accession no: E-MTAB-450.

* All reads combined for each strain which were used for solving the operon structures

Suppl. table S3. Comparison of mRNA half-lives as estimated by RT-qPCR, Illumina RNA-Seq (GA-II), and 454 RNA-Seq, for ten *B. cereus* ATCC 10987 genes used for method validation after normalization.

	RT-qPCR	GA-II	454
BCE_0020	1.3	1.9	2.3
BCE_0022	4.1	3.1	NA
BCE_0063	3.0	2.8	1.9
BCE_0267	1.4	1.6	1.6
BCE_0274	5.4	4.9	5.9
BCE_0351	5.1	4.3	NA
BCE_1421	3.4	3.3	NA
BCE_4389	11.5	13.9	4.9
BCE_4563	5.9	5.2	4.5
BCE_5615	1.2	2	NA

Suppl. table 4: Variables (numerical and non-numerical) tested for association with mRNA half-life[#]

Numerical factors:
**Gene expression (RPKM)**
CDS length
**CDS GC%**
**Number of genes in operon**
Length of 5'-UTR
Length of 3'-UTR
Space (nt) between RBS and CDS start [1]
Conservation (percent identity with orthologous genes in *B. cereus* ATCC 14579)
Number of predicted RNase binding sites
**Position (distance to origo)**
GC%, folding energy and length of secondary structure in 40 nt windows*
Number of single stranded nt at start and end of transcript
Non-numercial factors:
**COG classification** [2-4]
**KEGG classification** [5-7]
Gene groups (SigmaB, PlcR, NprR, two component systems, plasmid, phage, chromosome, strand) [8-10]
**Composition of RBS** [1]
**Repeat sequences present in transcript** [11, 12]
Core genome**
Gene strand (F/R)
Gene direction relative to the direction of replication***

[#]Factors significantly correlated with mRNA half-life are in bold face.

*40 first bases after TSS, 40 nt before or including RBS, 40 first nt of CDS, 40 nt after CDS stop, and 40 nt before TES were tested. The GC% of the first 40 nt after the TSS were significantly correlated with half-life.

** The core genome was defined as all proteins having an amino acid sequence identity of 85% or more to at least one protein in each of all other *B. cereus* group strains with closed genomes available at http://pathema.jcvi.org/cgi-bin/Bacillus/PathemaHomePage.cgi. The highly divergent *B. cereus* subsp. *cytotoxis* NVH 391-98 strain was excluded from the analysis. By these criteria 3109 ORFs were defined as the *B. cereus* core genome, similar to previous reports [13]. The pXO1-like core "genome" was defined similarly, except here the cut-off was set to 75% due to higher sequence divergence between these plasmids. Seventy-two genes were defined as the pXO1 core by these criteria.

*** Genes in the forward direction on the first half of the chromosome (0-180°) were defined as in line with the direction of replication, while genes in the reverse direction on the second half of the chromosome 180-360°) were defined as in line with the direction of replication.

1. Suzek, B.E., et al., *A probabilistic method for identifying start codons in bacterial genomes.* Bioinformatics, 2001. **17**(12): p. 1123-30.
2. Tatusov, R.L., et al., *The COG database: an updated version includes eukaryotes.* Bmc Bioinformatics, 2003. **4**: p. -.
3. Tatusov, R.L., et al., *The COG database: a tool for genome-scale analysis of protein functions and evolution.* Nucleic Acids Research, 2000. **28**(1): p. 33-36.
4. Tatusov, R.L., E.V. Koonin, and D.J. Lipman, *A genomic perspective on protein families.* Science, 1997. **278**(5338): p. 631-637.
5. Kanehisa, M., et al., *KEGG for representation and analysis of molecular networks involving diseases and drugs.* Nucleic Acids Res, 2010. **38**(Database issue): p. D355-60.
6. Kanehisa, M., et al., *From genomics to chemical genomics: new developments in KEGG.* Nucleic Acids Res, 2006. **34**(Database issue): p. D354-7.
7. Kanehisa, M. and S. Goto, *KEGG: kyoto encyclopedia of genes and genomes.* Nucleic Acids Res, 2000. **28**(1): p. 27-30.
8. Gohar, M., et al., *The PlcR virulence regulon of Bacillus cereus.* PLoS ONE, 2008. **3**(7): p. e2793.
9. van Schaik, W., et al., *Identification of the sigma(B) regulon of Bacillus cereus and conservation of sigma(B)-regulated genes in low-GC-content gram-positive bacteria.* Journal of Bacteriology, 2007. **189**(12): p. 4384-4390.
10. de Been, M., et al., *Comparative analysis of two-component signal transduction systems of Bacillus cereus, Bacillus thuringiensis and Bacillus anthracis.* Microbiology, 2006. **152**(Pt 10): p. 3035-48.
11. Tourasse, N.J., et al., *The Bacillus cereus group: novel aspects of population structure and genome dynamics.* Journal of Applied Microbiology, 2006. **101**(3): p. 579-593.
12. Økstad, O.A., et al., *Genome organization is not conserved between Bacillus cereus and Bacillus subtilis.* Microbiology-Sgm, 1999. **145**: p. 621-631.
13. Lapidus, A., et al., *Extending the Bacillus cereus group genomics to putative food-borne pathogens of different toxicity.* Chemico-Biological Interactions, 2008. **171**(2): p. 236-49.

Suppl. table S5. Factors correlated with mRNA expression level at t0 (*B. cereus* ATCC 10987).

Variable[a]	p-value	N	Correlation[b]
Half-life (min)	10^{-19}	2732	0.31
COG class	10^{-14}	2732	NA
KEGG pathway	10^{-16}	2733	NA
CG% coding region*	10^{-8}/0.02	2732/2333	0.31/0.16
Number of genes in operon **	10^{-45}/10^{-47}/10^{-24}	2732/2551/1940	0.48/0.39/0.24
Strand	10^{-5}	2732	For: 686 RPKM<br>Rev: 336 RPKM
Gene direction relative to the direction of replication	0.005	2732	S***: 611 RPKM<br>O: 134 RPKM
Chromosome (C) / Plasmid (P)	10^{-17}	2732	C: 516 RPKM<br>P: 55 RPKM
Position on chromosome (distance to origo)	10^{-19}	2735	-0.21
Degree of conservation	10^{-17}	2154	0.31
Core genome	10^{-13}	2735	Core: 641<br>Not core: 174
GC% of 40 first nt of 5 UTR	0.001	814	-0.11

[a] A description of the different variables is given in Suppl. table 4.

[b] The Pearson product moment correlation is given for numerical factors. For non-numerical factors of two variables the average expression values in RPKM are given.

*The two numbers given in each coloumn represent the data when: i) no normalization against GC% bias is used and ii) when the expression value was normalized for GC sequencing bias (according to the GC percentage). See manuscript text for details.

** The three numbers given in each coloumn represent the data when: i) all operons are used, ii) operons comprising more than 20 ORFs are omitted from the analysis, and iii) operons composed of more than 5 ORFs are omitted from the analysis.

*** S = Co-orientation with direction of replication fork movement, O = Opposite orientation relative to direction of replication fork movement.
