# Supplementary material for: Global mRNA decay analysis at single nucleotide resolution reveals segmental and positional degradation patterns in a Gram-positive bacterium
Source: Genome Biol. 2012 Apr 26;13(4):R30. doi: 10.1186/gb-2012-13-4-r30 (PMC3446304; doi:10.1186/gb-2012-13-4-r30)
Supplement: Additional file 2 — Supplementary Figures S1, S4, S5, S6, S7, S8, and S9. Supplementary Figure S1: distribution of the number of ORFs within operons. Supplementary Figure S4: statistical correlation (Pearson correlation) of calculated mRNA half-lives for the B. cereus ATCC 14579 B series and D series, respectively. Supplementary Figure S5: correlation between the RBS sequence and mRNA half-life. Supplementary Figure S6: correlation between mRNA expression level and selected factors. Supplementary Figure S7: expression of 16S rRNA at 2.5, 5, and 10 minutes after transcriptional arrest by rifampicin, relative to 16S rRNA expression at t(0) (time-point of rifampicin addition). Supplementary Figure S8: distribution of mapped read lengths from RNA sequencing using 454 technology. Supplementary Figure S9: explanation of the normalization procedure for RNA-Seq data using RT-qPCR. [file gb-2012-13-4-r30-S2.DOCX]

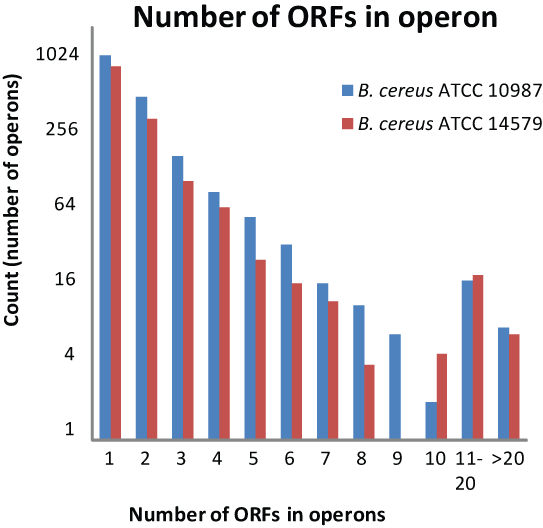


**Suppl. fig. S1.** Gene number distribution in operons from *B. cereus* strains ATCC 10987 and ATCC 14579, respectively (number of operons are drawn at log scale).


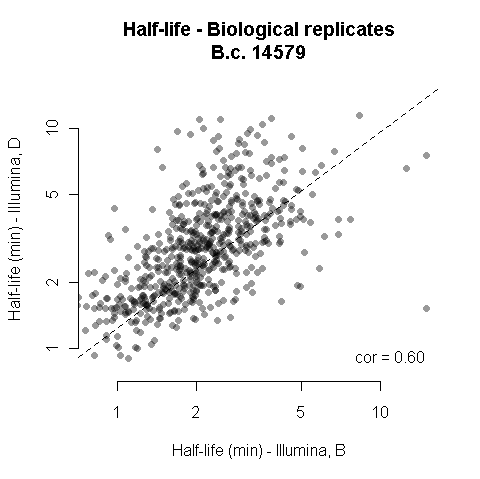


**Suppl. fig. S4.** Statistical correlation (Pearson correlation) of calculated mRNA half-lives from the two biological replicates (B-series and D-series, respectively) analyzed from *B. cereus* strain ATCC 14579.

**A B**


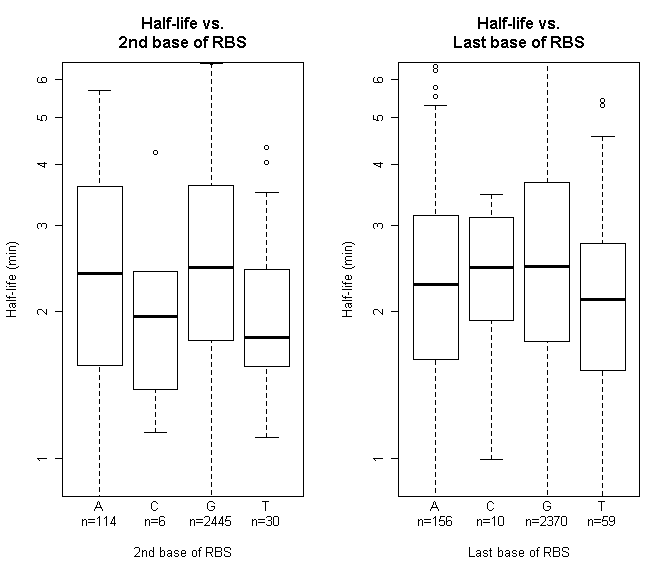


**C**


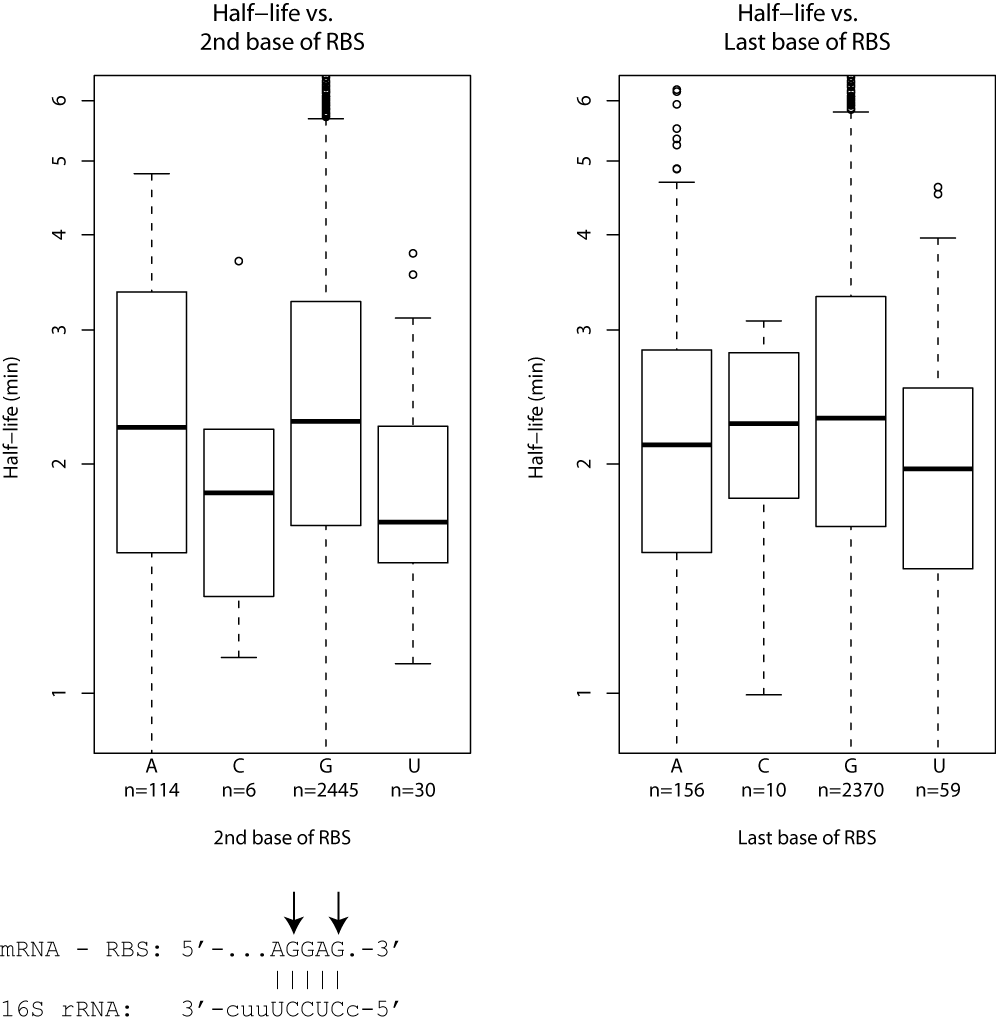


**Suppl. fig. S5.** Correlation between the base composition of the ribosomal binding sequence (RBS) (A: second base in RBS, B: Fifth base in RBS) and the half-life of its downstream gene in *B. cereus* ATCC 10987. The bottom panel (C) shows the base-pairing between the consensus RBS of an mRNA and the 16S rRNA of the *B. cereus* ribosome. The bases in the RBS which were analyzed are marked with arrows.


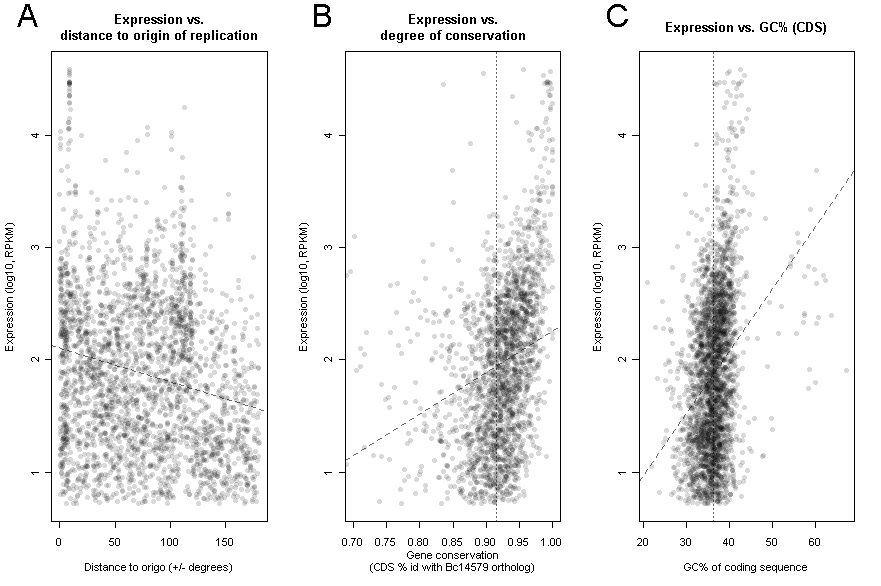


**Suppl. fig. S6.** Factors correlated with mRNA expression level of genes in *B. cereus* ATCC 10987 (A: distance to origo, B: gene conservation, C: GC% of coding sequence). Dashed lines represent regression lines for the correlation between the expression level and each tested factor. The vertical dotted lines in B and C represent the mean CDS id% (B) and GC% (C), respectively.

**Suppl. fig. S7.** Expression of 16S rRNA at 2.5, 5, and 10 minutes after transcriptional arrest by rifampicin, relative to 16S rRNA expression at t(0) (time point of rifampicin addition). 16S rRNA expression was measured by RT-qPCR in 12 replicate RNA decay series, using a constant amount of RNA from a constant amount of cells (in the absence of an internal reference), and experimental conditions as described in the Methods section. Furthermore, for the same samples, the average Pearson correlation of: 1) the amount of 16S rRNA at any one time point within a series, to 2) the length of time passed since rifampicin addition for that time point, was 0.07.


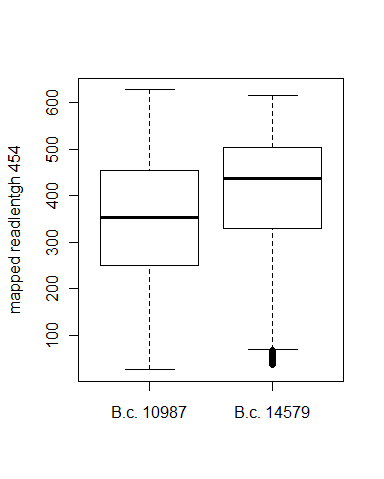


**Suppl. fig. S8.** Boxplot displaying the distribution of read lengths for reads acquired by 454 sequencing technology and which mapped uniquely to the genome, for *B. cereus* strains ATCC 10987 (B.c. 10987) and ATCC 14579 (B.c. 14579), respectively. Each box comprises the 25 to 75 percentile (i.e. the mid 50%) of mapped 454 read lengths. Remaining mapped read lengths (upper and lower 25 percentiles, respectively) are enclosed by whiskers, except for extreme outliers which are denoted by circles. The horizontal bar inside each box marks the median of mapped 454 read lengths for the two strains (354 bp for *B. cereus* ATCC 10987 and 438 bp for *B. cereus* ATCC 14579, respectively).


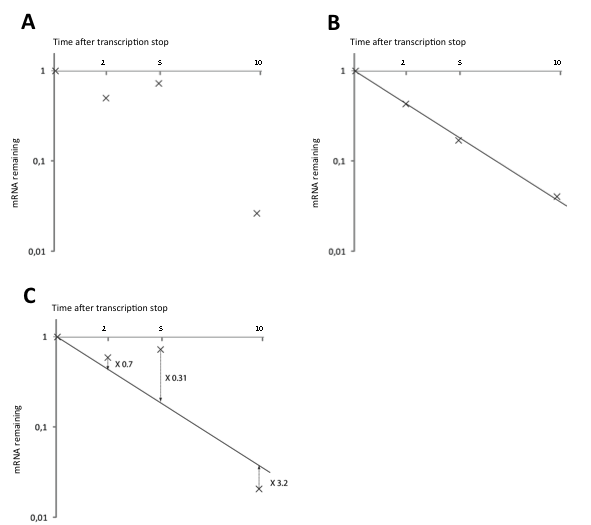


**Suppl. fig. S9**. Method for normalization of RNA sequencing data using quantitative real-time reverse transcriptase PCR (qPCR). Due to variable amounts of input RNA, and variable efficiency of cDNA synthesis as well as of RNA depletion in different samples, there was a need to normalize expression data in the RNA sequencing data set (example of RNA-Seq expression values for one gene for different time points in one biological replicate series is shown in panel A). Therefore, mRNA decay rates were determined for five chromosomal genes randomly selected from the *B. cereus* ATCC 14579 genome, by using RT-qPCR to calculate relative expression levels for each gene at all time points used in the mRNA decay analysis (example data for one gene is shown in panel B). A normalization factor was then calculated for each of the five genes, for each time point in the analysis (example for one gene is shown in panel C), by fitting the RNA-Seq data to the decay curve determined by RT-qPCR. For each time point, the RNA-Seq data for the rest of the genes in the genome was then normalized, using the geometric mean of the five normalization factors produced for the five genes.
