# Supplementary material for: Global mRNA decay analysis at single nucleotide resolution reveals segmental and positional degradation patterns in a Gram-positive bacterium
Source: Genome Biol. 2012 Apr 26;13(4):R30. doi: 10.1186/gb-2012-13-4-r30 (PMC3446304; doi:10.1186/gb-2012-13-4-r30)
Supplement: Additional file 4 — mRNA degradation patterns for the rbs (ribose utilization) operon in B. cereus ATCC 10987. Supplementary Figure S3 showing detailed mRNA decay patterns for the rbs operon. [file gb-2012-13-4-r30-S4.PDF]

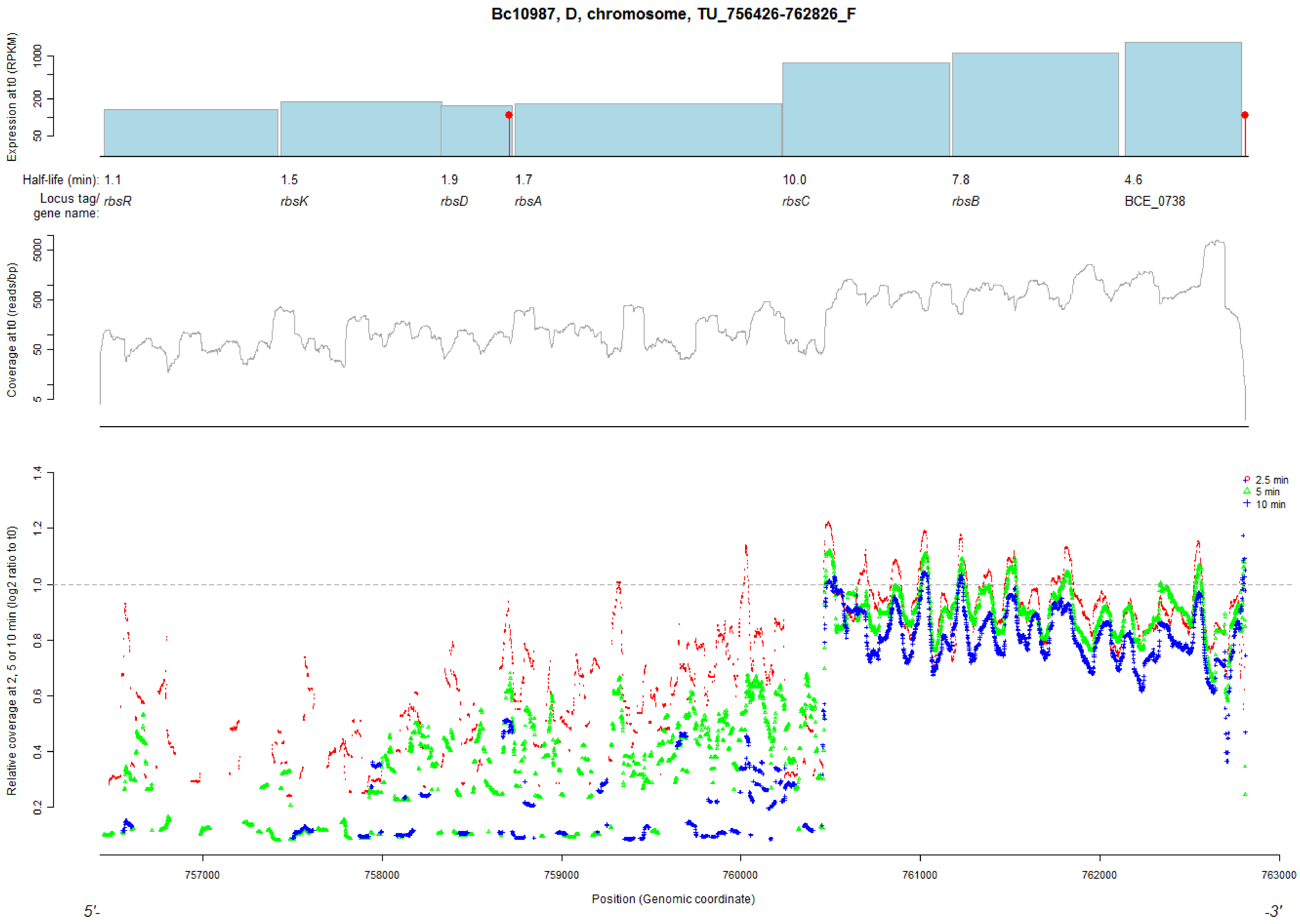


Suppl. fig. S3. Degradation patterns for the rbs (ribose utilization) operon in B. cereus ATCC 10987, analyzed and presented as for the gap operon in fig. 1. The first four genes, encoding a repressor (rbsR), a ribokinase (rbsK), and two subunits of an ABC transporter (including the ATP-binding subunit) (rbsD and rbsA), all had lower expression values (t0) and shorter half-lives than the three last genes of the operon, which encode the ABC transporter permease (rbsC) and ribose-binding (rbsB) subunits, and a putative transaldolase (BC0738).
