# Supplementary material for: Global mRNA decay analysis at single nucleotide resolution reveals segmental and positional degradation patterns in a Gram-positive bacterium
Source: Genome Biol. 2012 Apr 26;13(4):R30. doi: 10.1186/gb-2012-13-4-r30 (PMC3446304; doi:10.1186/gb-2012-13-4-r30)
Supplement: Additional file 5 — Global mRNA decay patterns at single nucleotide resolution. Detailed mRNA decay patterns of highly expressed operons. [file gb-2012-13-4-r30-S5.DOCX]

Additional file 5 can be downloaded from:

<http://folk.uio.no/aloechen/GenomeBiology/>

File name: Suppl_fig_S2_high_resolution_operons.pdf
